# Genotype-specific modulation of drought tolerance by arbuscular mycorrhizal symbiosis in foxtail millet

**DOI:** 10.3389/fpls.2025.1696600

**Published:** 2025-11-18

**Authors:** O-Chi Chang, Wei-Yi Lin

**Affiliations:** Department of Agronomy, National Taiwan University, Taipei, Taiwan

**Keywords:** arbuscular mycorrhizal fungi, symbiosis, drought, foxtail millet, transcriptome

## Abstract

Drought stress is a major environmental factor limiting crop productivity. Arbuscular mycorrhizal fungi (AMF), as beneficial soil microbes, can improve plant growth and stress resilience; however, the effectiveness of this symbiosis is often influenced by the host plant’s genetic background. In this study, we investigated the interaction between AM symbiosis and drought tolerance in two foxtail millet (*Setaria italica*) accessions with contrasting drought responses: the drought-tolerant ISE42 and the drought-sensitive TT8. Following a 14-day drought treatment, both accessions exhibited wilting, but AMF-colonized plants reduced malondialdehyde accumulation, indicating alleviated oxidative stress. Notably, only colonized ISE42 plants recovered upon rewatering. Although AMF colonization was confirmed by staining and qRT-PCR, AM symbiosis-conserved genes were strongly induced in ISE42 and TT8 only at 7 days post-treatment. Transcriptomic analysis further revealed that AM symbiosis significantly enhanced the expression of genes involved in nitrogen transport, assimilation, lignin metabolism, and cellulose biosynthesis in ISE42, suggesting improved nutrient uptake and cell wall reinforcement as key mechanisms underlying enhanced drought tolerance. In addition, drought-induced stress hormone signaling pathways were downregulated in colonized ISE42 roots, pointing to AM symbiosis-mediated stress alleviation. Together, these results demonstrate genotype-specific effects of AMF on drought tolerance and recovery capability, and highlight the importance of considering host genetic variation in the application of AMF for crop improvement.

## 1 Introduction

Climate change has led to a significant reduction in terrestrial water storage, contributing to the increased frequency and intensity of drought events, which in turn severely threaten global crop production and food security (Pokhrel et al., 2021). Under moderate drought conditions, yield losses can range from 30% to 90%, depending on the timing, duration and frequency of water deficit, and crop species involved (Vadez et al., 2024). To cope with drought stress, plants have evolved a range of adaptive mechanisms, including the enhancement of water uptake efficiency, minimizing water loss through stomatal closure, and mitigating oxidative stress via increased activities of anti-oxidative enzymes and accumulation of antioxidant compounds (Gupta et al., 2020).

Arbuscular mycorrhizal fungi (AMF) are soil-born microorganisms that establish symbiotic association with approximately 80% of terrestrial plant species. Through their external hyphal networks, AMF enhance the acquisition of water and mineral nutrients-especially phosphorus-from the soil and transfer them to host plants in exchange for photosynthetic products. The arbuscule, a highly branched fungal structure within root cortical cells, serves as the main site for nutrient exchange due to its large surface area (Smith and Smith, 2011; Kakouridis et al., 2022). Beyond nutrient acquisition, AMF colonization has been widely reported to confer increased tolerance to abiotic stress, including drought (Begum et al., 2019). Under drought conditions, AM symbiosis can induce osmolyte accumulation and enhance water and nutrient uptake efficiency, thereby alleviating osmotic stress in host plants, Furthermore, AMF modulate antioxidative enzyme activities, which help reduce the detrimental effects of oxidative stress during water deficit. AMF also influence abscisic acid (ABA) levels and ABA-mediated stress responses, ultimately supporting higher photosynthetic efficiency and biomass accumulation in colonized plants compared to non-mycorrhizal controls under drought stress (Liu et al., 2015; Chitarra et al., 2016; Ren et al., 2019; Nahuelcura et al., 2024).

Foxtail millet [*Setaria italica* (L.) P.Beauv.] is a small-grained cereal crop widely cultivated in arid and semi-arid regions in east Asia. As an ancient crop domesticated by various indigenous groups, foxtail millet exhibits significant genetic diversity. Morphological variation in plant height, flowering time, and panicle architecture has been observed across accessions collected from different continents (Ramesh et al., 2023; Xue et al., 2025). In Taiwan, foxtail millet is a staple food crop among indigenous communities, with over 300 landraces collected and grouped into three clusters based on molecular markers, which correspond to geographic distribution. These landraces exhibit variation in agronomic traits and starch content, indicating high genetic diversity within the Taiwanese germplasm (Lin et al., 2012; Kuo et al., 2018). Compared to other C4 plants such as maize and sorghum, foxtail millet possesses a dense, deep root system and thickened cell walls, contributing to its high water-use efficiency and remarkable drought tolerance (Terfa et al., 2025). Notably, its yield remains largely unaffected by drought stress occurring after heading (Matsuura et al., 2012). Comparative transcriptomic studies of drought-tolerant and -sensitive accessions have identified candidate genes involved in metabolic pathways, stress signaling, gluconeogenesis, transcriptional regulation, and proteolysis that are associated with drought adaptation in foxtail millet (Zhang et al., 2007; Lata et al., 2010; Shi et al., 2018; Xu et al., 2019).

The role of AMF in alleviating drought stress has been validated in multiple crop species, including soybean, citrus, maize, and foxtail millet (Gong et al., 2015; Begum et al., 2019; Cheng et al., 2022; Oliveira et al., 2022). Although foxtail millet is generally considered drought tolerant, genetic variation in drought responses has been documented among accessions (Mohammadi-Nejad et al., 2017; Vaezi et al., 2020). Moreover, increasing evidence indicated that the outcome of AMF symbiosis on plant growth and nutrient acquisition is strongly influenced by both host and fungal genotypes (Mateus et al., 2019; Watts-Williams et al., 2019; Chang and Lin, 2023). Despite this, limited is known about the difference in drought tolerance among host genotypes affect the efficacy of AMF symbiosis under drought condition.

In this study, we investigated the AMF-mediated modulation of drought stress responses in foxtail millet by comparing a drought-tolerant and a drought-sensitive accession. We evaluated growth performance and physiological responses under drought stress with and without AMF colonization and performed transcriptomic analysis to uncover molecular mechanisms underlying the genotype-dependent effects of AM symbiosis. These findings provide insights into the interaction between host genetic background and AMF symbiosis and have potential implication for improving drought resilience in foxtail millet through targeted microbial and genetic interventions.

## 2 Materials and methods

### 2.1 Plant materials and growth conditions

Plants were cultivated in a growth chamber under a 12-hour light (28°C)/dark (22°C) photoperiod with a light intensity of approximately 200 μmol m^-2^ sec^-1^ and 60% relative humidity. In this study, a drought-sensitive accession, TaiTung8 (TT8), and a high drought-tolerant accession, ISE42, were selected for investigation. Seeds were pre-treated at 45°C dry bath for 30 minutes and surface-sterilized with 2% NaOCl. Two weeks after germination, seedlings were transplanted into sterile cones filled with a 9:1 (v/v) sterilized substrate mixture of river sands and peat moss.

*Claroideoglomus etunicatum*, provided by Dr. Jui-Chang Huang (Tainan District Agricultural Research and Extension Station, Taiwan) was used as the AMF inoculant. Foxtail millet was employed as the host plant for propagating fungal inoculum. For each plant, 10 mL of inoculum containing approximately 1000 spores were applied to the substrate before transplanting. All plants were watered daily with equal volumes of water and fertilized biweekly with a nutrient solution (N: P_2_O_5_: K_2_O = 15: 5: 25; Cheng et al., 2022) until initiation of drought stress treatment.

At six weeks post-transplanting, irrigation was withheld for two weeks to induce drought stress. Watering was resumed for four days to assess plant recovery. Plants were harvested at 7 and 14 days after the onset of drought, and at 4-days after rewatering, for evaluating stress responses and RNA isolation.

### 2.2 AMF staining and evaluation of colonization efficiency

Mycorrhizal roots were fragmented and cleaned in 10% (w/v) KOH at 90°C for 30 minutes. Subsequently, the roots were transferred to 0.3 N HCl for 30 minutes for neutralization. Next, roots were stained with 0.1% (w/v) trypan blue overnight, followed by de-staining with acidic glycerol (Phillips and Hayman, 1970).

Colonization efficiency was quantified using the gridline intersect method as described by Mcgonigle et al. (1990). Over 100 root fragments were randomly selected and examined under an Olympus SZX-16 stereomicroscope (Olympus, Japan). Colonization efficiency (%) was calculated using following formula:


(Numberof colonized root intersections/ Total number of root intersections)×100%.

### 2.3 Malondialdehyde concentration measurement

Around 0.1g leaf tissue was homogenized in 4 mL of 5% (w/v) trichloroacetic acid (TCA) and centrifuged at 10,000 ×*g* for 10 minutes. The supernatant (1 mL) with 4 mL of 0.5% thiobarbituric acid (TBA) in 5% TCA. The reaction mixture was incubated at 95°C for 30 minutes, cooled on ice, and centrifuged at 10,000 ×*g* for 10 minutes at 4°C. The absorbance was measured at 532 nm (specific) and at 600 nm (non-specific) using a spectrophotometer (Heath and Packer, 1968). MDA concentration was calculated using the extinction coefficient of 155 mM^-1^cm^-1^ and expressed as nanomole per gram of fresh weight (nmole g^-1^ FW).

### 2.4 Catalase and ascorbate peroxidase activity evaluation

Around 0.1g leaf tissue was homogenized in 4 mL of 50 mM potassium phosphate buffer (pH 6.8) and centrifuged at 12,000 ×*g* for 20 minutes at 4°C. Catalase (CAT) activity was determined by mixing 0.2 mL of the resulting supernatant with 2.7 mL of 100 mM sodium phosphate buffer (pH 7.0) and 0.1 mL of 1 M H_2_O_2_. The change of absorbance at 240 nm was recorded for 1 minute, following the method described by (Kato and Shimizu, 1987). One unit of CAT activity is defined as 1 nmol of H_2_O_2_ consumed per minute. Ascorbate peroxidase (APX) activity was measured by adding 0.1 mL of supernatant to a reaction mixture containing 1 mL of 150 mM potassium phosphate buffer (pH 7.0), 1 mL of 150 mM ascorbate, 0.4 mL of 0.75 mM EDTA, and 0.5 mL of 6 mM H_2_O_2_. The change of absorbance at 290 nm was monitored for 1 minute. One unit of APX activity is defined as 1 μmol ascorbate consumed per minute (Nakano and Asada, 1981).

### 2.5 RNA isolation and gene expression analysis

Total RNA was extracted following the method by Wang and Vodkin (1994). Briefly, 100 mg of ground root tissues was homogenized in 0.7 mL extraction buffer containing 4% SDS, 20 mM DTT, 78 mM Tris, 17.2 mM EDTA-Na_2_ and 156 mM NaCl. RNA was subsequently purified using phenol: chloroform: isoamylalcohol (25:24:1). Two steps of RNA precipitation were carried out using 4M LiCl and isopropanol with 3M NaOAc, respectively. The resulting RNA pellet was dissolved in nuclease-free water and stored at -80°C until further use. Three replicates were prepared for each treatment.

Genomic DNA contamination was removed using 5× gDNA Eraser (Tools, Taiwan), and 500 ng of total RNA was used for first-strained cDNA synthesis using Moloney murine leukemia virus reverse transcriptase (Thermo Fisher Scientific, USA). Quantitative real-time PCR (qRT-PCR) was performed using iQ™ SYBR^®^ Green Supermix (Bio-Rad, USA) on a CFX Connect Real-Time PCR Detection System (Bio-Rad). Relative gene expression was normalized to the reference gene *SiEF1α* and was expressed as 2^-ΔCt^. The primers used in this study are listed in **Supplementary Table 1**.

### 2.6 RNA sequencing and analysis

The quantity and quality of RNA was assessed using a SimpliNano™ spectrophotometer (Biochrom, USA). For RNA sequencing, 1 μg of RNA per sample was used for library preparation. RNA libraries were prepared using the KAPA mRNA HyperPrep Kit (Roche, Switzerland) in combination with KAPA Pure Beads system (Roche) for fragment sorting. Sequencing was conducted on a Illumina NovaSeq 6000 platform (Illumina, USA).

High quality reads were selected and the adapter sequences were trimmed using Trimmomatic v0.38 (Bolger et al., 2014). The reads were aligned to the *Setaria italica* reference genome v2.0 (Bennetzen et al., 2012) using HISAT2 v2.1.0 (Kim et al., 2015). Relative gene expression levels and identification of differentially expressed genes (DEGs) were analyzed using edgeR v3.28.1 and DESeq2 v.1.26.0, respectively (Anders et al., 2013; Love et al., 2014). DEGs were defined based on a false discovery rate (FDR) threshold of< 0.05 and ∣log_2_ fold change (FC)∣ ≥ 1. Gene ontology (GO) enrichment of DEGs was performed using clusterProfiler v3.14.3 R package (Yu et al., 2012).

### 2.7 Statistical analysis

All data were analyzed using R software. Difference among treatments were assessed by analysis of variance (ANOVA), followed by Tukey’s honest significant difference (HSD) test at a significance level of *p* < 0.05.

## 3 Results

### 3.1 Variation of drought responses and AM symbiotic effects between two millet accessions

To investigate whether symbiotic effects on drought tolerance in foxtail millet vary by genotype, we used two contrasting accessions: TT8, a drought-sensitive accession, and ISE42, a highly drought-tolerant accession. These genotypes were examined for their dynamic responses to drought and the influence of AMF colonization.

**Figure 1:**
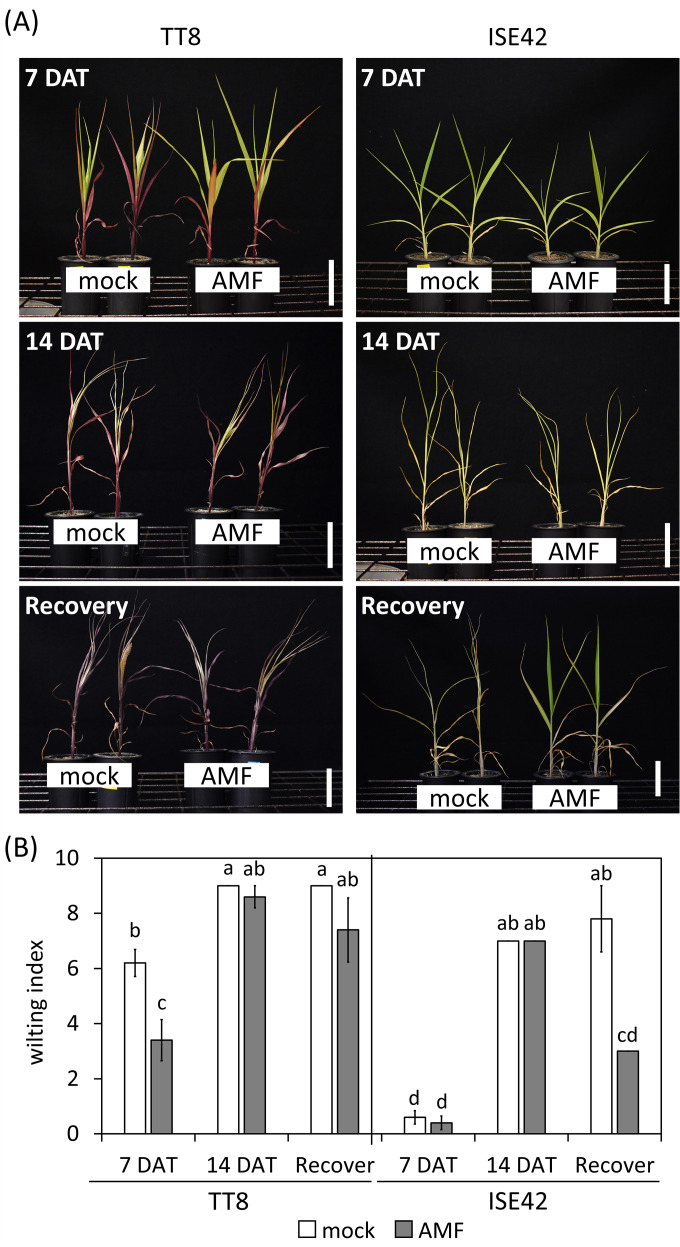
Morphology and wilting index of TT8 and ISE42 during drought stress and after recovery. **(A)** Morphology of mock- and AMF-treated TT8 and ISE42 at 7 and 14 DAT and 4 days after water resupply. **(B)** Wilting index of plants. N = 5. Error bars represent the standard error of the mean. Data were analyzed using ANOVA (*p* < 0.05) followed by Tukey’s HSD test. Different letters above the bars indicate statistically significant differences.

At seven days after drought treatment (7 DAT), mock-treated TT8 plants exhibited erect leaves, whereas AMF-inoculated TT8 plants showed drooping leaves (**Figure 1A**). Although no significant differences in shoot or root fresh weight were observed between treatments at this stage, the wilting index was significantly lower in AMF-inoculated TT8 compared to the mock control (**Figure 1B**). In contrast, ISE42 plants maintained normal growth and showing no wilting symptoms, regardless of AMF inoculation (**Figure 1**).

**Figure 2:**
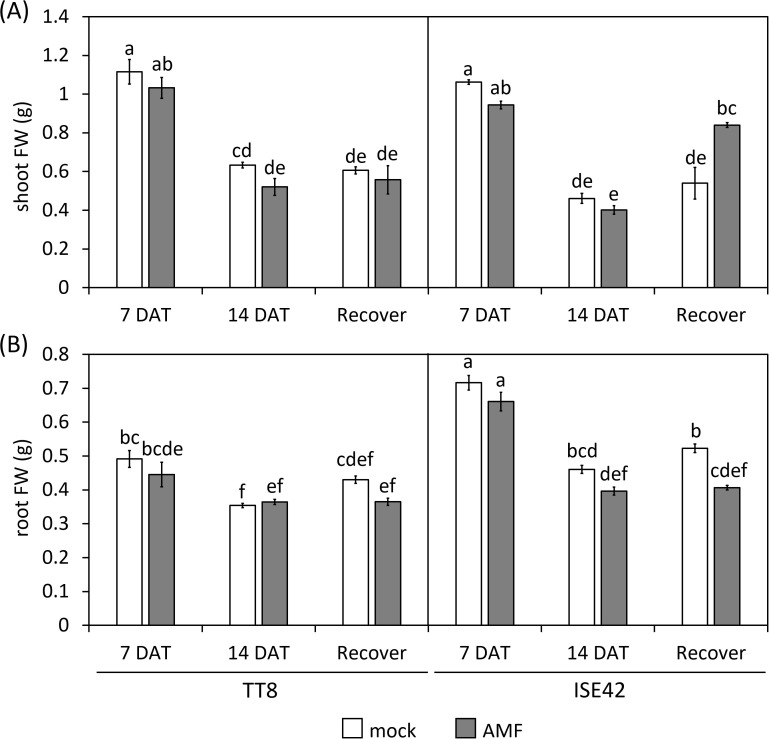
Shoot **(A)** and root **(B)** fresh weight of TT8 and ISE42 during drought stress and after recovery. FW, fresh weight. N = 5. Error bars represent the standard error of the mean. Data were analyzed using ANOVA (*p* < 0.05) followed by Tukey’s HSD test. Different letters above the bars indicate statistically significant differences.

By 14 DAT, both accessions exhibited severe growth retardation and wilting. AMF colonization did not fully prevent stress symptoms in either genotype. Upon rewatering for four days, most TT8 plants remained wilted, irrespective of AMF colonization. Conversely, AMF-inoculated ISE42 plants recovered and displayed green, turgid leaves, unlike mock-treated ISE42 plants with remained wilted (**Figure 1**). These phenotypes were reflected in shoot fresh weight measurement: ISE42 displayed a significant increase in shoot biomass after rehydration. In contrast, the shoot biomass in TT8 gradually declined under drought and remained low after rehydration, regardless of AMF treatment. Root fresh weight decreased under drought in both accessions but did not recover following rehydration (**Figure 2**).

AMF staining indicated similar colonization efficiency between the two accessions, with no significant change under drought conditions. Based on the relative expression of *C. etunicatum β-Tubulin* gene, the AMF colonization in ISE42 at 7 DAT was highest, whereas TT8 showed no noticeable effects by prolonged drought treatment (**Supplementary Figure 1**), Taken together, the AMF colonization efficiency and host physiological responses suggested that the differential drought responses between the two accessions are unlikely to be due to variation in AMF colonization efficiency.

**Figure 3:**
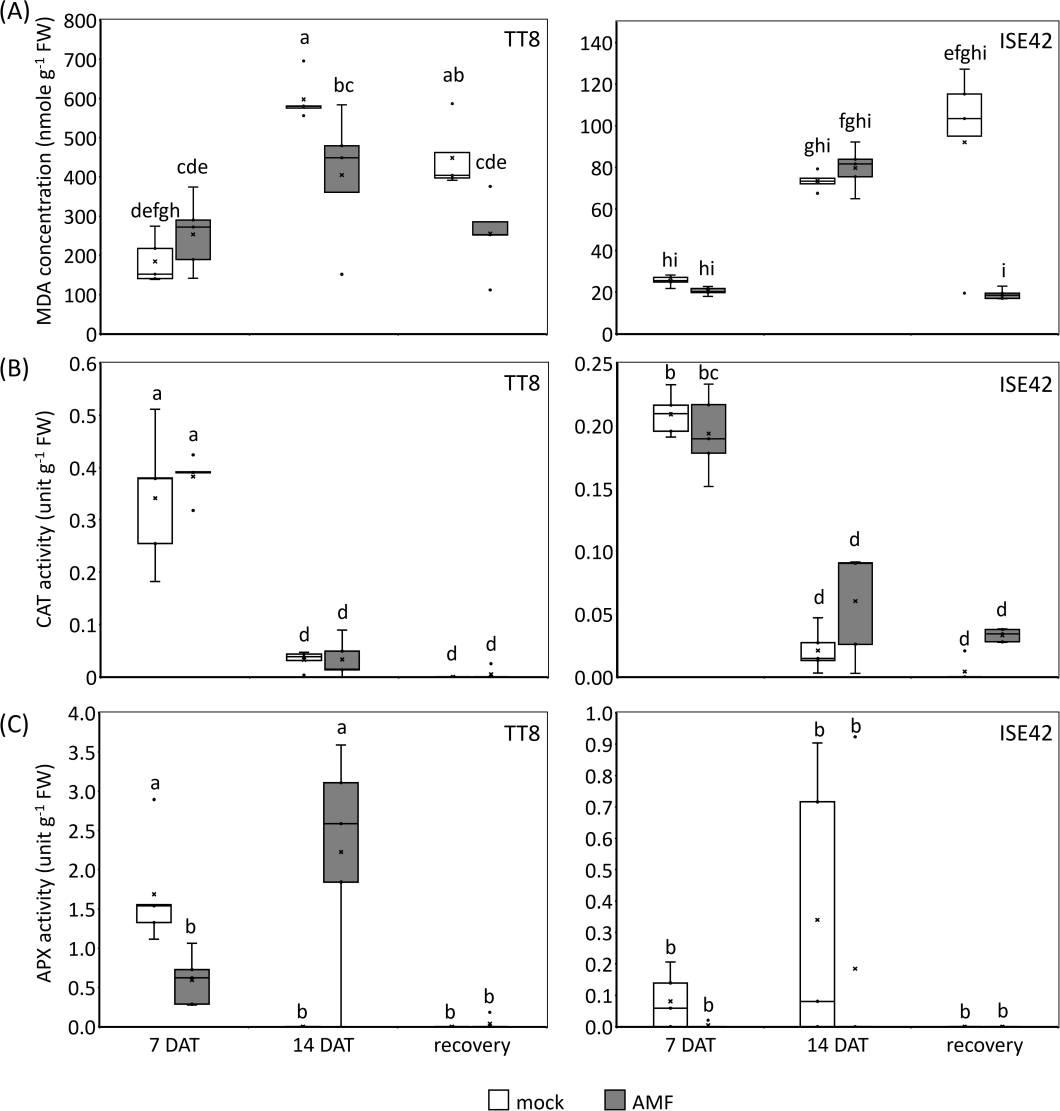
Indicators of oxidative stresses in TT8 and ISE42 during drought stress and after recovery. **(A)** Malondialdehyde (MDA) concentrations, **(B)** catalase (CAT) activity, and **(C)** ascorbate peroxidase (APX) activity. The horizontal lines within boxes indicate median values, the upper and bottom boundaries represent the 25^th^ and 75^th^ percentiles. N = 5. Data were analyzed using ANOVA (*p* < 0.05) followed by Tukey’s HSD test. Different letters above the bars indicate statistically significant differences.

During water deficit conditions, photosynthesis efficiency is declined due to stomata closure, leading to reactive oxygen species (ROS) accumulation (Cruz de Carvalho, 2008). To assess oxidative stress and ROS scavenging activity, we measured malondialdehyde (MDA)-a marker of lipid peroxidation-and the activities of two antioxidant enzymes: catalase (CAT) and ascorbate peroxidase (APX). Prolonged drought treatment increased MDA levels in both accessions; however, ISE42 accumulated significantly less MDA than TT8, consistent with its shoot phenotype. At 7 DAT, no significant differences in MDA content were observed between AMF and mock treatments. By 14 DAT, MDA levels significantly decreased in AMF-inoculated TT8 compared to the mock control. Following rehydration, MDA content in mock-treated plants remained high, whereas AMF-colonized plants in both accessions showed a significant reduction in MDA levels (**Figure 3A**), indicating that AMF symbiosis alleviated oxidative stress.

In both accessions, CAT activity peaked at 7 DAT but declined sharply at 14 DAT and remained low after rehydration. No significant differences in CAT activity were observed between AMF and mock treatments in either accession (**Figure 3B**). APX activity in ISE42 remained low throughout drought and recovery, with no apparent effect from AMF colonization. In contrast, in TT8, APX activity increased progressively under drought in AMF-treated plants, while it was undetectable in mock-treated plants by 14 DAT (**Figure 3C**). The inverse relationship between MDA levels and APX activity in TT8 suggests that APX may play a critical role in mitigating oxidative damage during drought in AMF-colonized plants.

### 3.2 Dynamics of transcriptome during drought stress treatments

To elucidate the genome-wide effects of AM symbiosis on drought stress responses in the two accessions, transcriptomic analyses were performed on roots of mock- and AMF-treated plants at 7 and 14 DAT. These analyses aimed to capture the dynamic expression profiles of stress- and symbiosis-responsive genes.

After trimming and filtering, each RNA-seq library yielded between 3.4 × 10^7^ and 6.4 × 10^7^ high-quality reads, with Q_30_ values exceeding 93% (**Supplementary Table 2**). More than 78.2% of reads were successfully mapped to the foxtail millet reference genome and over 75.4% represented uniquely mapped reads (**Supplementary Table 3**). To validate the transcriptomic data, ten differentially expressed genes (DEGs) were selected for qRT-PCR analysis. The high correlation coefficient (R^2^) between RNA-seq and qRT-PCR results confirmed the reliability of the transcriptomic analysis (**Supplementary Figures 2**, **3**).

**Figure 4:**
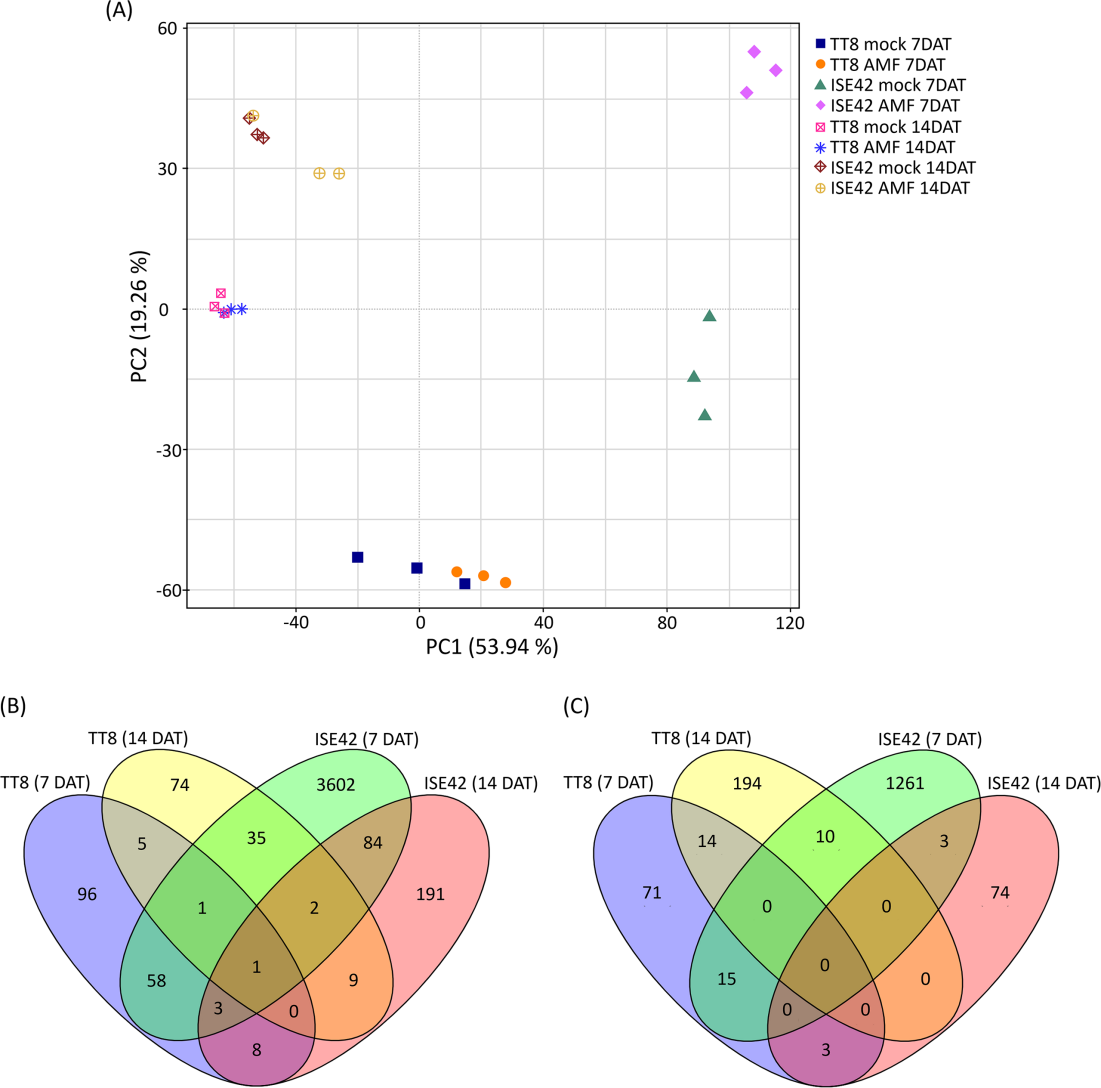
Overview of transcriptome analysis. **(A)** Principal component analysis (PCA) of transcriptomic data. **(B, C)** Venn diagrams of upregulated and downregulated genes in response to AM symbiosis, respectively.

Principle Component Analysis (PCA) was conducted to assess the consistency of biological replicates and to explore global expression variation across treatments. PC1 and PC2 accounted for 53.9% and 19.3% of the total variance, respectively. Replicates clustered tightly within each treatment group, while samples from TT8 and ISE42 were clearly separated, indicating distinct transcriptomic responses to drought between the two accessions (**Figure 4A**). Notably, clear separation between mock- and AMF-treated samples was observed at 7 DAT, particularly in ISE42. However, by 14 DAT, transcriptomic profiles of mock- and AMF-treated samples converged in both accessions, suggesting that prolonged drought diminished the transcriptional impact of AM symbiosis (**Figure 4A**).

DEGs were identified using fold change were >2 or<0.5 with FDR< 0.05. In TT8, 172 genes were upregulated and 103 downregulated at 7 DAT, while 127 genes were upregulated and 218 downregulated at 14 DAT. In contrast, ISE42 exhibited a much stronger transcriptional responses at 7 DAT, with 3786 upregulated and 1289 downregulated genes. However, at 14 DAT, only 298 and 80 genes were up- and downregulated, respectively, consistent with the reduced symbiotic effect observed in PCA results.

Comparative DEG analysis 63 commonly upregulated and 15 commonly downregulated genes between TT8 and ISE42 in response to AM symbiosis at 7 DAT. At 14 DAT, only 12 genes were commonly upregulated and none downregulated between two accessions. Temporal comparisons within the same genotype showed minimal overlap in TT8 (7 and 14 genes up- and downregulated at both time points, respectively), whereas ISE42 displayed 90 commonly upregulated and 3 commonly downregulated genes across 7 and 14 DAT (**Figures 4B,C**). These results suggested that AMF colonization elicited a stronger and more dynamic transcriptional responses in drought-tolerant accession ISE42 compared to the drought-sensitive TT8.

### 3.3 Gene ontology analysis of DEGs

To gain an overview of the functional roles of DEGs, Gene Ontology (GO) enrichment analysis was conducted. Given the high number of DEGs in ISE42 at 7 DAT, 20 and 8 Biological Property (BP) GO terms were significantly enriched among up- and down-regulated genes, respectively. In contrast, only 6 and 11 BP terms were enriched in up- and down-regulated genes in TT8, respectively.

Consistent with the observed beneficial effects of AM symbiosis on drought tolerance in ISE42, upregulated genes were significantly enriched in GO terms associated with ROS detoxification. These included hydrogen peroxide catabolic/metabolic process (GO:0042744, GO:0042743), cellular detoxification (GO:1990748, GO:0098754, GO:0098869), reactive oxygen species metabolic process (GO: 0072593), oxylipin metabolic/biosynthesis process (GO:0031407, GO:0031408) and peroxidase activity (GO:0004601). Interestingly, the GO term “response to hydrogen peroxide (GO:004252)” was enriched in downregulated genes. Additionally, genes involved in jasmonic acid (JA)-mediated signaling pathway (GO:0009867), which is known to play a role in stress responses, were upregulated in ISE42 (**Supplementary Table 4**).

Unexpectedly, several GO terms related to water and fluid transport (GO:0006833, GO:0042044, GO:0005372, GO:0015250) and metal ion transport (GO:0030001, GO:0005506) were enriched in genes downregulated by AM symbiosis in ISE42 at 7 DAT (**Supplementary Table 5**). These findings suggested that AM symbiosis may contribute to drought tolerance in ISE42 not only through ROS detoxification but also by modulating transport process to optimize water and ion balance.

In contrast, responses to AM symbiosis in TT8 was characterized by enrichment of GO terms related to cellular responses to phosphate starvation/nutrient levels (GO:0016036, GO:0009267, GO:0031667, GO:0031669), which are the classical markers of symbiotic responses. Despite a lower wilting index in AM-colonized TT8 plants, no GO terms directly related to drought stress were significantly enriched, implying that AMF may enhance drought performance in TT8 primarily through improved nutrient acquisition, particularly phosphate (**Supplementary Tables 4**, **5**).

At 14 DAG, despite several visible dehydration symptoms, GO enrichment analysis identified 12 and 1 BP terms among upregulated and downregulated genes, respectively, in TT8. Among upregulated terms, several were associated with phenylpropanoid biosynthesis and metabolic pathways (GO:0009698, GO:0009699), cinnamic acid biosynthesis/metabolic process (GO:0009800, GO:0009803) and phenylalanine catabolic/metabolic process (GO:0006558, GO:0006559), all of which are involved in the production of phenolic compounds that help mitigate abiotic stress. Meanwhile, responses to hydrogen peroxide (GO:0042542) were enriched in downregulated genes, suggesting delayed activation of oxidative stress mitigation pathways by AM colonization in TT8 (**Supplementary Tables 4**, **5**).

In ISE42 at 14 DAT, 16 BP terms were enriched among DEGs regulated by AM symbiosis. Notably, more than 75% of downregulated BP terms were associated with cell wall biosynthesis. Conversely, upregulated DEGs were enriched in terms related to lignin metabolism (GO:0046274, GO:0019748, GO:0009808). These results suggested that under prolonged drought, AMF may enhance drought tolerance in ISE42 through the modulation of cell wall biosynthesis and remodeling, which could contribute to improve mechanical strength and stress resilience (**Supplementary Tables 4**, **5**).

### 3.4 Genotype-dependent regulation of AM symbiosis-conserved genes under drought stress

Genes essential for AM symbiosis are widely conserved across host plant genomes (Delaux et al., 2014; Favre et al., 2014; Bravo et al., 2016). To assess the influence of drought stress on these genes and compared genotype-specific responses, we examined the expression patterns of 63 AM symbiosis-conserved genes in TT8 and ISE42. At 7 DAT, 44 of these genes were significantly upregulated in ISE42, indicating strong transcriptional activation of the symbiotic pathway under drought conditions. In contrast, only three genes were significantly induced in TT8, despite previous reports of widespread upregulation under well-watered conditions (Chang and Lin, 2023). By 14 DAT, induction of symbiosis-conserved genes was markedly reduced or undetectable in both accessions (**Supplementary Table 6**). Staining confirmed the presence of fungal structures in roots (**Supplementary Figure 1**), yet drought reduced plant-fungus interaction. These findings indicated that transcriptional activation of AM symbiosis-conserved genes under drought stress is strongly genotype-dependent, and that prolonged drought suppresses the expression of these genes, suggesting a dominant effect of drought over AM symbiosis on the root transcriptome.

### 3.5 Differential regulation of aquaporin genes by AM symbiosis under short-term and prolonged drought stress

**Figure 5:**
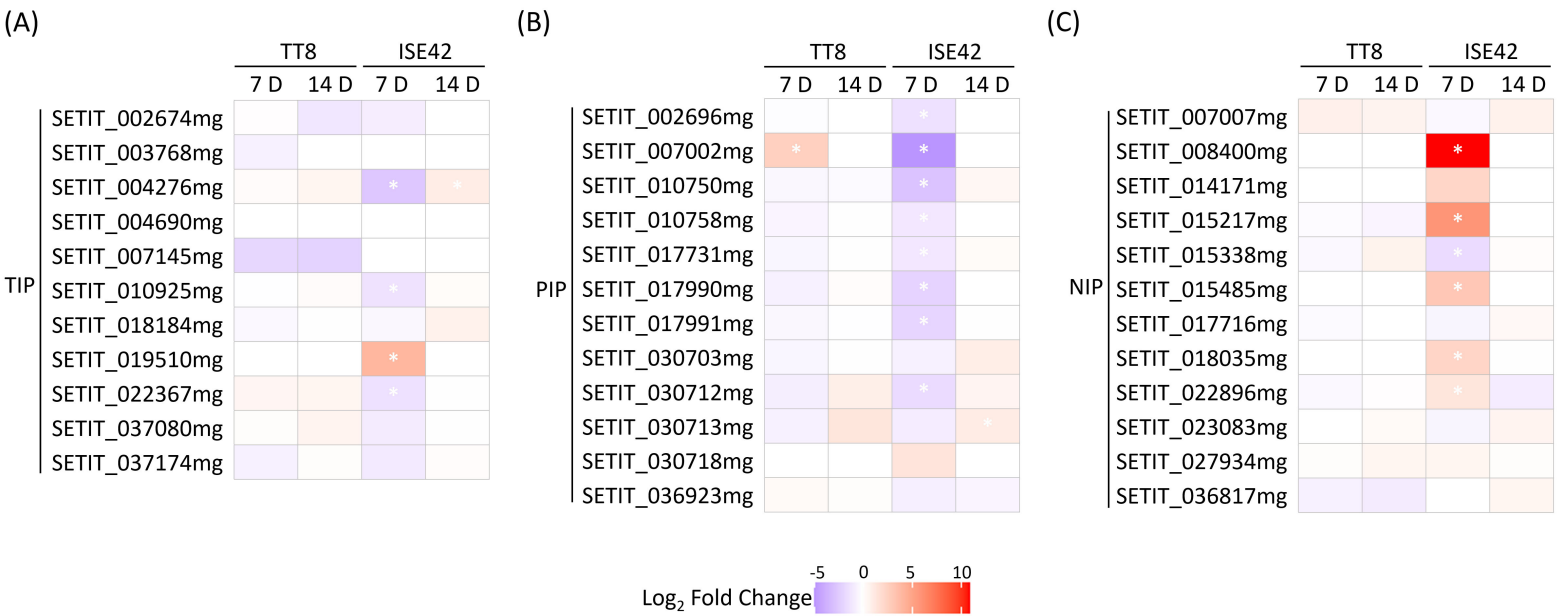
Differentially expressed aquaporin genes in TT8 and ISE42 during drought stress in response to AM symbiosis. **(A-C)** members of the TIP, PIP and NIP subfamilies. Asterisks (*) indicates statistically significant differences.

Aquaporins (AQPs), particularly members of the plasma membrane intrinsic protein (PIP) and tonoplast intrinsic protein (TIP) families, play critical roles in facilitating water uptake under drought stress (Shivaraj et al., 2021), and their regulation by AM symbiosis can vary with drought severity (Bárzana et al., 2014). In this study, we investigated how the interaction between plant genotype and AM symbiosis influenced *AQP* expression under both short-term and prolonged drought conditions. At 7 DAT, most of *PIP* and *TIP* genes in TT8 were either unaffected or slightly downregulated by AM symbiosis, whereas in ISE42, AMF colonization markedly suppressed the expression of these genes (**Figures 5A,B**), suggesting more effective drought alleviation in ISE42. Notably, several *NOD26-like intrinsic protein* (*NIP*) genes were upregulated in AMF-colonized ISE42 at 7 DAT, but remained unchanged in TT8 (**Figure 5C**). By 14 DAT, expression differences between AMF-inoculated and mock-treated plants were negligible in both accessions (**Figure 5**), indicating that the symbiotic modulation of *AQP* expression is largely confined to early drought responses.

### 3.6 Regulation of nutrient transport genes by AM symbiosis under drought

**Figure 6:**
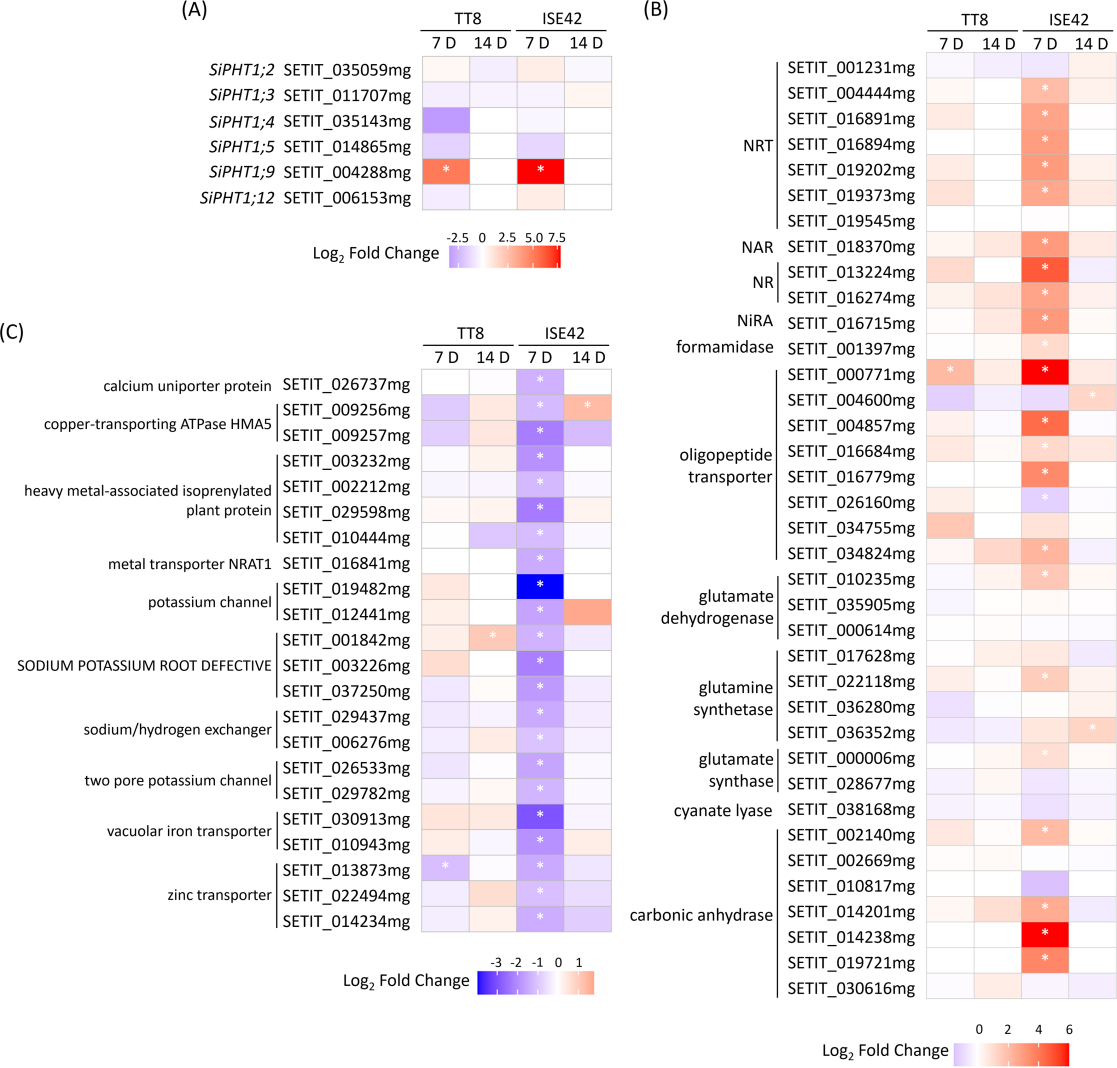
Differentially expressed genes associated with nutrient uptake and assimilation during drought stress in response to AM symbiosis. **(A)** Phosphate transporter genes. **(B)** Nitrate transporter and nitrogen assimilation genes. **(C)** Metal ion transporter genes. Asterisks (*) indicate statistically significant differences.

AM symbiosis is known to enhance plant nutrient uptake efficiency, particularly for phosphate and nitrogen (Wang et al., 2017). To assess genotype-specific responses under drought, we examined nutrient transporter gene expression in TT8 and ISE42. Of the 12 annotated phosphate transporter 1 (PHT1) family members, only six homologs were detected. Consistent with previous reports (Ceasar et al., 2014; Chang and Lin, 2023), *PHT1;9*, a AM symbiosis-conserved gene, was strongly upregulated at 7 DAT in both accessions, but its expression became undetectable at 14 DAT, likely due to prolonged drought suppression. The remaining five *PHT1* genes were either downregulated or unaffected by AM colonization (**Figure 6A**).

Nitrogen transport and assimilation showed pronounced genotype-specific regulation. At 7 DAT, five nitrate transporter (*NRT*) genes were significantly upregulated in colonized ISE42 roots, while TT8 showed no significant change. In ISE42, genes encoding nitrate reductase (NR), nitrite reductase (Nir), and carbonic anhydrase—supplying carbon skeletons for nitrogen assimilation—were also induced, along with glutamine synthase and glutamate synthase, key enzymes in primary amino acid biosynthesis (**Figure 6B**). These effects were absent in TT8 and diminished by 14 DAT, suggesting that enhanced nitrogen acquisition and assimilation may contribute to the improved drought recovery in ISE42.

In contrast, several metal ion channels and transporters, including potassium channels, iron transporters, calcium transporter and zinc transporters, were downregulated by AM symbiosis in ISE42 at 7 DAT. In TT8, only a few metal transporter genes were affected by symbiosis (**Figure 6C**), potentially reflecting a shift in ion homeostasis or prioritization of essential nutrient uptake under symbiosis and drought.

### 3.7 AM symbiosis modulates phytohormone signaling pathways under drought stress in a genotype-specific manner

**Figure 7:**
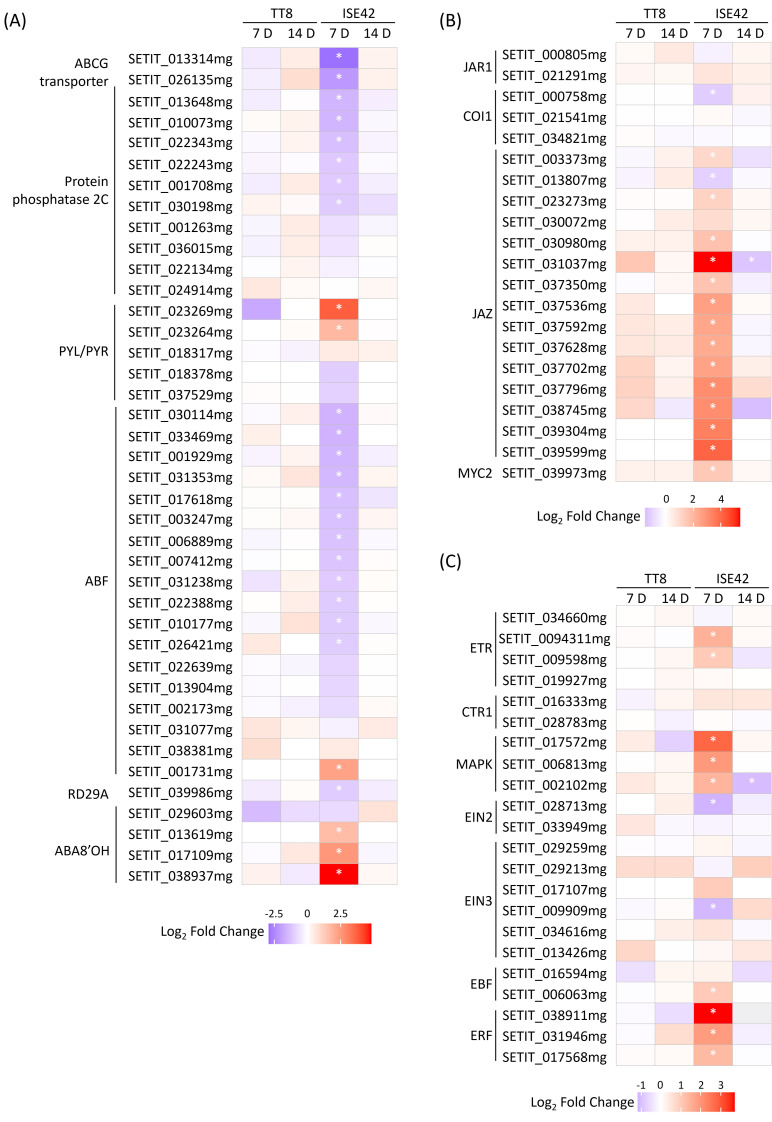
Differentially expressed genes involved in hormone signaling during drought stress in response to AM symbiosis. **(A-C)** ABA, JA and ethylene signaling genes. Asterisks (*) indicate statistically significant differences.

The role of abscisic acid (ABA) in regulating plant drought stress responses has been well-established (Aslam et al., 2022). In ISE42 at 7 DAT, AM colonization upregulated two of five *PYL/PYR*, encoding ABA receptors, and genes encoding protein phosphatase 2C (PP2C), the negative regulators of ABA signaling were downregulated. However, several *ABA-responsive element-binding factor* (*ABF*) genes and *Response to Desiccation* (*RD*) *29* (Jia et al., 2012) were repressed, while *ABA 8’-hydroxylase* (*ABA8’OH*) genes, encoding ABA catabolic enzymes, were induced. Additionally, homologs of *AtABCG25* and *MtABCG20* homologs, encoding a root-to-shoot ABA transporter and an ABA exporter in roots, respectively, was also reduced by symbiosis (**Figure 7A**). The changes suggest fine tuning of ABA perception and transport. By 14 DAT, expression differences were no longer apparent. In TT8, AM colonization had minimal effects on ABA signaling at either time points (**Figure 7A**).

GO enrichment indicated that JA-mediated signaling pathways were also modulated by AM symbiosis. Although JA is traditionally associated with biotic stress responses, it also plays a critical role in coordinating drought responses, partly by enhancing ABA accumulation (Balbi and Devoto, 2008; Liu et al., 2016). At 7 DAT, several *JAZ* genes, encoding JA repressors, were upregulated in AMF-colonized ISE42, while *JAR1* and *COI1*, which are involved in JA signal perception were either unaffected or slightly repressed, suggesting a general suppression of JA signaling in ISE42 (**Figure 7B**). Despite the repression of JA signaling genes, genes involved in α-linolenic acid metabolism, which contributes to JA biosynthesis, and *MYC2* gene, a key JA-responsive transcriptional regulator were upregulated (**Figure 7B** and **Supplementary Figure 4**). No differences were detected by 14 DAT.

Ethylene (ET), another stress-related hormone, is known to positively influence drought tolerance (Gusain et al., 2024). In ISE42, AM colonization upregulated multiple components of ET signaling pathway at 7 DAT, including two ET receptors, three MAP kinases and two ET responsive transcription factors (ERFs). TT8 showed no significant changes (**Figure 7C**).

Collectively, these results highlight a complex, genotype-specific modulation of phytohormone signaling by AM symbiosis in ISE42, characterized by suppression of ABA and JA signaling components alongside activation of ethylene signaling pathways, potentially contributing to enhanced drought tolerance.

### 3.8 Genotype-specific activation of transcription factors and calcium signaling pathways by AM symbiosis under drought stress conditions

Members of NAC or APETALA2/ethylene-responsive element binding factors (AP2/ERF) transcription factor families are key regulators of abiotic stress tolerance, including drought (Wang and Dane, 2013; Ma et al., 2024). Transcriptomic profiling identified 88 *AP2/ERF* and 13 *DREB* genes across treatments. In ISE42 roots, AM symbiosis induced 27 *AP2/ERF* and 3 *DREB* genes at 7 DAT and 4 *AP2/ERF* and 5 *DREB* genes at 14 DAT. No induction was observed in TT8; instead, one and three genes were downregulated at 7 and 14 DAT, respectively (**Supplementary Table 7**). Among 106 NAC-domain containing genes detected, 28 were specifically upregulated in colonized ISE42 at 7 DAT, whereas only one and two genes were induced in ISE42 and TT8, respectively, at 14 DAT (**Supplementary Table 7**). These results indicate strong genotype-specific activation of NAC and AP2/ERF transcription factors in AMF-mediated drought responses.

Calcium functions as a secondary messenger in mediating diverse cellular responses (Rudd and Franklin-Tong, 2001). CALCIUM-PERMEABLE STRESS-GATED CATION CHANNEL1 (CSC1) mediates calcium fluxes under osmotic stress (Hou et al., 2014; Maity et al., 2019). Of 12 *CSC1* homologs identified, four and one were significantly upregulated in ISE42 roots at 7 DAT and 14 DAT, respectively, under AM symbiosis, with no induction in TT8. Calcium signals are decoded by calcium-dependent protein kinase (CDPK), which activate downstream signaling cascades (Dekomah et al., 2022). Of 29 foxtail millet *CDPK* genes, 25 were expressed under at least one treatment, and 11 were specifically induced by AM symbiosis in ISE42 roots at 7 DAT (**Supplementary Table 8**). These finding suggest that enhanced drought tolerance in ISE42 may be mediated through CDPK-dependent calcium signaling pathways.

### 3.9 AM symbiosis enhanced phenylpropanoid and cell wall biosynthesis pathway in a genotype-specific manner under drought stress

The phenylpropanoid biosynthesis converts phenylalanine into aromatic compounds, including flavonoids and lignin, which are critical for oxidative and drought stresses (Vogt, 2010; Sharma et al., 2019). Arogenate dehydratase (ADT), the rate-limiting step in phenylalanine biosynthesis pathways, was strongly induced by AM symbiosis in ISE42 at 7 DAT, with four out of five *ADT* homologs upregulated, compared to only one in TT8.

**Figure 8:**
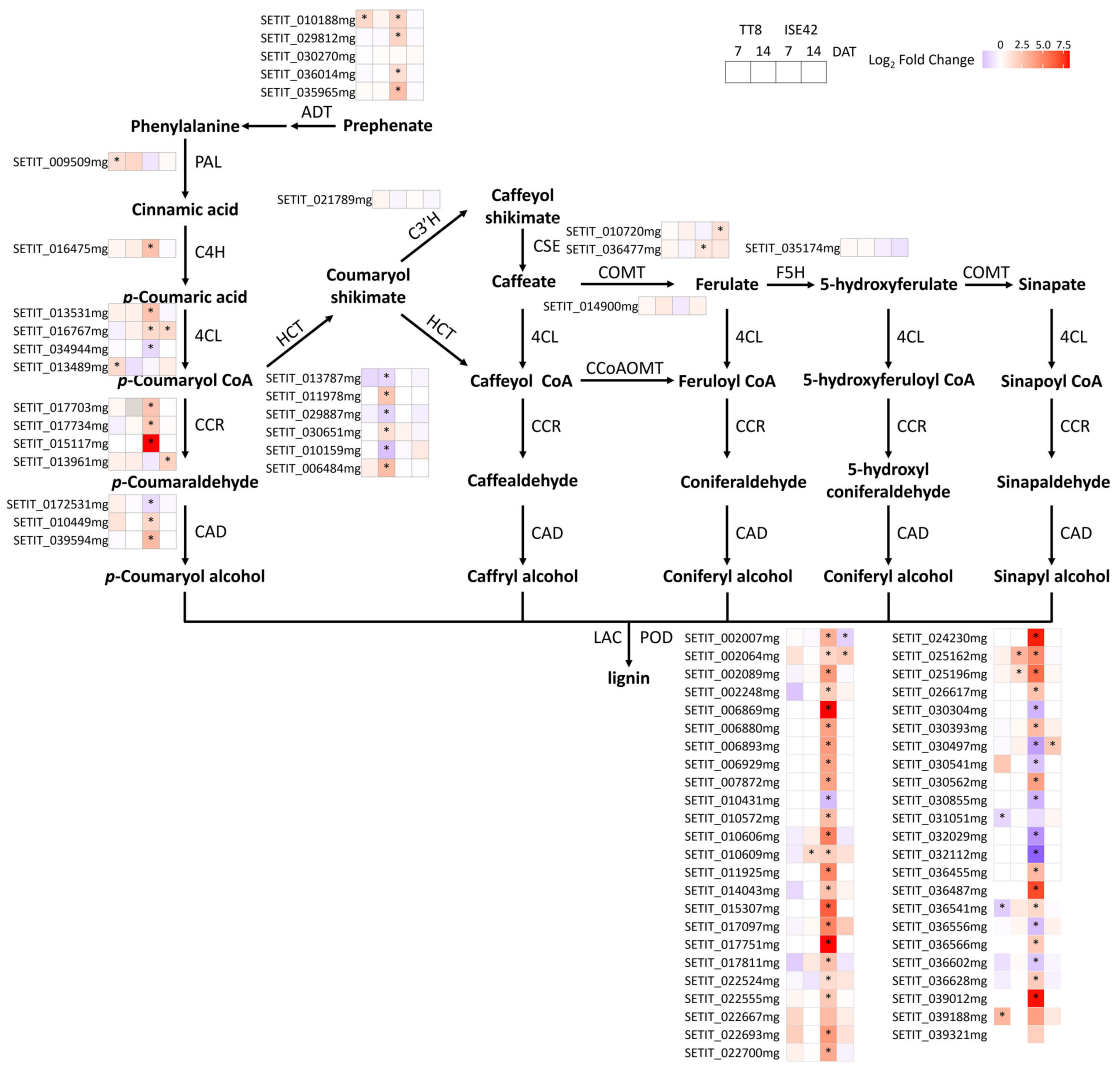
Differentially expressed genes in phenylpropanoid pathways during drought stress in response to AM symbiosis. Asterisks (*) indicate statistically significant differences.

In the phenylpropanoid biosynthesis pathway, several key genes encoding *4-coumarate:CoA ligase* (*4CL*), *cinnamoyl-CoA reductase* (*CCR*) and *cinnamyl-alcohol dehydrogenase* (*CAD*) were significantly upregulated in colonized ISE42 roots at 7 DAT. In TT8, only one *phenylalanine ammonia-lyase (PAL*) gene and one *4CL* gene showed induction. Peroxidases (PODs), responsible for oxidative polymerization of monolignols during lignin biosynthesis, were also strongly upregulated in ISE42, with over 30 *POD* genes induced by AM symbiosis, while induction in TT8 was negligible (**Figure 8**).

Genes associated with cell wall biosynthesis, including *cellulose synthases* (*CESAs*) and *COBRA* genes were also affected by AM symbiosis. At 7 DAT, six *CESA* genes and two *COBRA* genes were upregulated in colonized ISE42, compared with only one *CESA* and one *COBRA* in TT8. At 14 DAT, GO terms related to cell wall biosynthesis and metabolism were enriched among upregulated genes in TT8, including several *CESA* and *laccase* genes, with a similar late-stage pattern also observed in ISE42.

Collectively, these results indicate that AM symbiosis promotes lignin biosynthesis and cell wall remodeling primarily in ISE42, potentially strengthening root structure and enhancing drought tolerance, with both genotypes. This symbiosis-driven modification likely facilitates root adaptation to prolonged drought stress, underscoring a genotype-dependent response mechanism.

## 4 Discussion

### 4.1 Genetic variation shapes AMF-mediated drought responses

Genetic variation in host plants is a key determinant of AM symbiotic responses, as reported in sorghum, cassava, barley, maize and foxtail millet (Mateus et al., 2019; Watts-Williams et al., 2019; Al Mutairi et al., 2020; Chang and Lin, 2023; Li et al., 2023). Our previous work showed that most AM symbiosis-conserved genes in TT8 were upregulated under low-phosphate conditions; however, in the present study, their expression declined in TT8 after seven days of drought stress, whereas ISE42 maintained stable expression. Similar studies in maize and sorghum demonstrated a positive correlation between AMF compatibility, nutrient homeostasis and growth responses (Watts-Williams et al., 2019; Li et al., 2023). Nevertheless, ecological studies suggested that symbiotic effects on plant growth are not universally positive and depend on plant-fungus combination (Klironomos, 2003).

Here, AMF colonization efficiency did not differ between genotypes or between short- and long-term drought stress, indicating similar establishment of symbiosis. However, the marked differences in the expression of symbiosis-conserved genes and drought-responsive genes between accessions highlight significant genotype × environmental interactions. Together with previous findings and our results suggest that the reprogramming of both symbiosis-conserved and non-conserved genes under drought stress is strongly genotype-dependent. Notably, AMF association substantially enhanced drought tolerance in ISE42, whereas only minor improvements were observed in TT8, reinforcing the impact of host genetic variation on AMF-mediated drought responses.

### 4.2 AM symbiosis-mediated regulation of aquaporin genes under drought stress conditions

Enhancement of water uptake and plasma membrane-anchored *AQP*s (*PIP*s) expression by AM symbiosis under drought stress has been reported in many plant species, supporting the role of PIPs in water permeability and transport (He et al., 2019; Wang et al., 2023; Wang, D. Y. et al., 2024; Zou et al., 2024; Ni et al., 2025). However, our results, along with other studies (Chitarra et al., 2016; Porcel et al., 2006; Quiroga et al., 2017; Symanczik et al., 2020), reveal that AMF association can also lead to downregulation of certain *PIP* genes under water deficit conditions. This pattern may reflect stress severity- and duration-dependent regulation, where *PIP*s induced by AM symbiosis during mild drought are subsequently suppressed under prolonged stress to reduce water loss (Bárzana et al., 2014; Porcel et al., 2006). Indeed, PIP downregulation has been correlated with increased leaf relative water content, suggesting a role in water conservation (Porcel et al., 2006 and Aroca et al., 2007).

In contrast, several *NIP*s were specifically induced in colonized ISE42. In *Lotus japonica*, LjNIP1;5 enhances drought tolerance and AMF maintenance by promoting root proliferation and reducing stomata aperture (Zou et al., 2024). The foxtail millet homolog (SETIT_018035mg) was similarly induced in colonized ISE42 but not in TT8, coinciding with stronger activation of symbiosis-conserved genes. These findings suggest that NIP-type proteins may stabilize plant-fungus interactions, potentially through transport of water or small molecules within arbuscule-containing cells.

Host and fungal genotypes also influence *AQP* expression (Quiroga et al., 2017; Symanczik et al., 2020). While some studies reported greater AMF benefits in drought-sensitive cultivars (Quiroga et al., 2017), our results showed stronger advantages in the tolerant ISE42, possibly due to differences in host and fungal species used. Such context-dependent variation emphasizes the need for broader genotype-genotype testing to understand symbiotic modulation of water transport mechanisms.

### 4.3 Impacts of AM symbiosis on nitrogen uptake and assimilation

Drought-induced stomata closure limits both transpiration and nitrogen assimilation, restricting photosynthesis and growth (McDowell et al., 2008; Robredo et al., 2011; Bista et al., 2018; Xia et al., 2020; Han et al., 2022). Exogenous application of nitrogen source can enhance water use efficiency, nitrogen assimilation, and antioxidative activities, alleviating water deficit stress (Brueck et al., 2010; Ren et al., 2015; Meng et al., 2016; Li et al., 2020; Pissolato et al., 2020; Ren et al., 2020). Furthermore, adequate nitrogen supply promotes drought recovery capacity (Sergent et al., 2014; Maywald et al., 2022; Sun et al., 2025), highlighting the importance of maintaining N supply and assimilation for drought tolerance.

AM symbiosis facilitates uptake of phosphorus, nitrogen and other nutrients in host plants. In rice, approximately 40% of root-acquired N is delivered via OsNPF4.5-mediated symbiotic route, with additional symbiosis-induced NRT/NPF family members functioning under low nitrate conditions (Wang et al., 2020). In ISE2, homologs of *OsNPF.5* (SETIT_004857mg), *OsNRT2.3a* (root-to-shoot transport; SETIT_004444mg)) (Tang et al., 2012), and *OsNPF8.1* (organic N mobilization in response to drought; SETIT_000816mg) (Qiu et al., 2023) were upregulated by AM symbiosis, but not in TT8. This suggests enhanced acquisition from soil and fungal partners, plus efficient root-to-shoot nitrogen mobilization, supports drought resilience.

Drought also suppresses nitrate reduction (Gloser et al., 2020), partly via drought and salt tolerance (DST)-mediated downregulation of nitrate reductase genes in rice (Han et al., 2022). Conversely, nitrate supplementation can increase nitrate reductase activity and nitric oxide (NO) production, enhancing antioxidant capacity (Pissolato et al., 2020). In ISE42, AM symbiosis upregulated nitrate reductase, nitrite reductase, glutamine synthase and glutamate synthase genes, but not nitric oxide synthase (data not shown), indicating improved N assimilation contributes to its superior drought tolerance.

### 4.4 AM symbiosis-mediated drought tolerance via the enhancement of lignin biosynthesis and cellulose biosynthesis

After 7 days of drought treatment, AMF-colonized ISE42 roots showed strong induction of lignin biosynthesis genes. Lignin provides structural support and facilitates water transport through xylem, thereby contributing to drought resistance (Weng and Chapple, 2010; Ménard et al., 2022). In rice, *OsCCR10*, encoding a cinnamoyl-coA reductase in the monolignol biosynthesis pathway, is induced by OsNAC5 in roots under drought and its overexpression increased root lignin content, reduces water loss, and enhances drought tolerance (Jeong et al., 2013; Bang et al., 2022). Similarly, OsNAC17 and drought-responsive AP2/ERF transcription factors such as OsERF71 and sweet potato RAP2.4 enhance drought tolerance via activation of lignin biosynthesis genes (Lee et al., 2016; Bian et al., 2022). In ISE42, AM symbiosis enhanced the expression of genes of for monolignol biosynthesis and polymerization, suggesting that lignin accumulation contributes to symbiosis-mediated drought tolerance. In contrast, colonized TT8 displayed minimal activation of these pathways, aligning with its weaker symbiotic transcriptional responses. Prolonged drought suppressed lignin-related gene induction in both genotypes.

In addition to lignin, cellulose—a key structural component of the cell wall—plays an important role in osmotic stress tolerance. Loss-of-function mutations in CESA and CESA-like proteins impair polysaccharide deposition and ROS regulation, reducing osmotic tolerance (Zhu et al., 2010; Hou et al., 2024). In our study, more CESA and COBRA-lie genes were upregulated in colonized ISE42 than in TT8 at both 7 and 14 DAT, indicating that strong symbiotic responses in ISE42 may enhance cellulose biosynthesis and confer greater drought resistance.

### 4.5 Regulation of ABA and JA homeostasis by arbuscular mycorrhizal symbiosis in drought responses

ABA plays a central role in plant drought responses, not only in regulating stomata aperture but also in modulating root growth and angle under water stress (Feng et al., 2022; Xiong, Y. L. et al., 2025). In our study, *ABA8’OH* genes were upregulated in colonized ISE42 at 7 DAT, coinciding with reduced expression of *ABF*s and *RD29A.*This pattern was absent in TT8, suggesting that AM symbiosis in ISE42 may mitigate drought-induced ABA signaling, possibly by alleviating stress perception. Such trends are consistent with previous findings in wheat, where drought-sensitive cultivars exhibited strong ABA accumulation, whereas tolerant cultivars showed elevated *ABA8’OH* expression without significant ABA accumulation (Ji et al., 2011). In tomato, AMF colonization under drought condition similarly reduced ABA accumulation (Chitarra et al., 2016; Sánchez-Romera et al., 2018). Notably, in maize, exogenous ABA application enhanced drought tolerance, yet this effect was attenuated in AMF-colonized plants, with reduced aquaporin expression compared to controls (Ruiz-Lozano et al., 2009). Together, these results suggest that AM symbiosis promotes drought adaptation not through sustained ABA accumulation, but through fine control of ABA homeostasis.

JA signaling also exhibited distinct modulation under AM symbiosis in ISE42. The upregulation of *JAZ* genes, which encode negative regulators of JA responses, indicates a repression of canonical JA signaling. This repression is known to reduce drought tolerance in JAZ-overexpressing plants and mutants (Fu et al., 2017; Wang, W. M. et al., 2024; Xiong, J. Y. et al., 2025). However, we also observed upregulation of α-linolenic acid metabolism genes, critical for JA biosynthesis, along with *MYC2*, a master activator of JA signaling. This suggests that AM symbiosis may employ a fine-tuning strategy, simultaneously tempering excessive JA responses via JAZs while maintaining basal JA-mediated defenses through increased biosynthesis and *MYC2* activation. Such a mechanism may help balance energy allocation between growth and stress defense.

The interplay between JA and ABA may further contribute to AM-mediated drought responses. JA has been shown to act upstream of ABA by repressing PP2C, thereby enhancing osmotic stress tolerance (Zhao et al., 2023). Additionally, JA transport from roots to shoots can influence shoot-level responses such as stomatal regulation and transpiration control (Ogawa et al., 2021). The observed increase in JA biosynthesis in AM-colonized ISE42 could therefore support both local and systemic to reprogram hormone signaling networks, integrating ABA and JA pathways to optimize drought adaptation. Further work will focus on characterizing shoot-level molecular and physiological responses to clarify the full extent of this regulatory integration.

### 4.6 Conclusion

This study demonstrates clear genotype-dependent variation in AMF-mediated drought responses in foxtail millet. The drought-tolerant ISE42 displayed pronounced transcriptomic activation of nutrient transport, cell wall reinforcement and hormonal fine-tuning, leading to improved drought resilience. In contrast, TT8 showed limited transcriptional and physiological benefits. These results underscore the complexity of plant-fungus interaction and highlight the need to consider host genotype when applying AMF in crop drought management strategies.

## Data Availability

The datasets presented in this study can be found in online repositories. The names of the repository/repositories and accession number(s) can be found below: https://www.ncbi.nlm.nih.gov/, GSE306689.
