# Supplementary material for: Genotype-specific modulation of drought tolerance by arbuscular mycorrhizal symbiosis in foxtail millet
Source: Front Plant Sci. 2025 Nov 18;16:1696600. doi: 10.3389/fpls.2025.1696600 (PMC12668936; doi:10.3389/fpls.2025.1696600)
Supplement: Supplementary file 1 [file DataSheet1.pdf]

frontiers

Supplementary Material

Supplementary Table 1. Sequences of primers used in this study for qRT-PCR analyses.

Accession	primer	Sequence
SETIT_000254mg	qSi000254_F	TGCAGAATGAAGCACCCACT
	qSi000254_R	TTAGCCACTCCAGCAACCTG
SETIT_004153mg	qSi004153_F	GTCGGAAGAGATTGCACGGA
	qSi004153_R	ATACAATGAGCCGCAGCCAA
SETIT_009413mg	qSi009413_F	TCGAGCTATGCTACGCACTG
	qSi009413_R	GAACAGGGCCAATGGGATGA
SETIT_016794mg	qSi016794_F	CCGTAGCCGAGACCTACATTC
	qSi016794_R	GGACGGAGACGAGCACATAG
SETIT_016682mg	qSi016682_F	ACACCACCTTCATCCAGCAG
	qSi016682_R	GCCAAACGAGAATCCGCATC
SETIT_020982mg	qSi020982_F	TGGTACGGGCAAGAGGTAAG
	qSi020982_R	AGGAAGGAAGGTGCCACAAT
SETIT_021174mg	qSi021174_F	GAACTGAGGCAGAAGGCACT
	qSi021174_R	GGGGTCCAGAAGCCGAATAG
SETIT_022040mg	qSiEF1a_F	CAACAAGATGGATGCCACCAC
	qSiEF1a_R	GAGATTGGGACGAAGGCAATC
SETIT_029251mg	qSi029251_F	GGAGATGACCTCGGACGTTG
	qSi029251_R	GCCGCACTTGATGAGGTACT
SETIT_030712mg	qSi030712_F	AAGCACTCACCTTCCCAACA
	qSi030712_R	GACAATGCAAGCTCTCGCTG
SETIT_037147mg	qSi037147_F	CGTCCCTAGTGTTCCTCCCT
	qSi037147_R	ATCTGCTTCTTGAGCCGCTT
C. *etunicatum* β-*Tubulin* (FJ174279.1)	qCeTub_F	ACACAGTACCATTCCCACGTC
	qCeTub_R	CGGTCAATGCACGGTATCCT

Supplementary Table 2. The quantity and quality of reads.

Sample	Raw Read	Clean Read	Q20 (%)	Q30 (%)	GC content (%)
mock7D TT8_1	53763406	47675182	98.74	95.305	53.04
mock7D TT8_2	51863662	46084272	98.745	95.32	53.055
mock7D TT8_3	59921894	52773004	98.775	95.39	52.74
AMF7D TT8_1	65402166	57583320	98.725	95.25	52.22
AMF7D TT8_2	60103564	53453330	98.815	95.515	52.3
AMF7D TT8_3	52257474	45846912	98.705	95.17	51.52
mock7D ISE42_1	73547240	64382594	98.795	95.44	52.175
mock7D ISE42_2	65446612	57080448	98.77	95.395	52.385
mock7D ISE42_3	59038434	52595648	98.795	95.435	52.33
AMF7D ISE42_1	69662944	61369528	98.785	95.415	52.835
AMF7D ISE42_2	60307380	53260916	98.735	95.27	52.485
AMF7D ISE42_3	53839920	48390380	98.82	95.505	51.985
mock14D TT8_1	52973140	44041358	98.275	94.005	53.96
mock14D TT8_2	52727852	39566824	98.265	93.965	51.28
mock14D TT8_3	51977368	43030582	98.305	94.07	53.73
AMF14D TT8_1	53428698	44935916	98.33	94.13	52.89
AMF14D TT8_2	58082476	46755688	98.225	93.85	52.42
AMF14D TT8_3	42034220	34965366	98.27	93.965	52.6
mock14D ISE42_1	41723240	35084668	98.28	93.985	53.715
mock14D ISE42_2	50093852	42346422	98.32	94.1	53.83
mock14D ISE42_3	51045832	43055688	98.32	94.08	53.665
AMF14D ISE42_1	43546972	36494082	98.265	93.95	53.61
AMF14D ISE42_2	47955500	40322494	98.31	94.04	53.015
AMF14D ISE42_3	51190748	42785656	98.395	94.32	53.875

Supplementary Table 3. The percentage of reads mapped to the genome.

Type	Total Reads	Total Mapped	Multiple Mapped	Uniquely Mapped
mock7D TT8_1	47675182 (100 %)	45130891 (94.7 %)	1839151 (3.9 %)	43291740 (90.8 %)
mock7D TT8_2	46084272 (100 %)	43712873 (94.9 %)	1918720 (4.2 %)	41794153 (90.7 %)
mock7D TT8_3	52773004 (100 %)	50060989 (94.9 %)	1877206 (3.6 %)	48183783 (91.3 %)
AMF7D TT8_1	57583320 (100 %)	52761899 (91.6 %)	2071706 (3.6 %)	50690193 (88.0 %)
AMF7D TT8_2	53453330 (100 %)	48728481 (91.2 %)	1922656 (3.6 %)	46805825 (87.6 %)
AMF7D TT8_3	45846912 (100 %)	40125974 (87.5 %)	1640096 (3.6 %)	38485878 (83.9 %)
mock7D ISE42_1	64382594 (100 %)	61524669 (95.6 %)	3029367 (4.7 %)	58495302 (90.9 %)
mock7D ISE42_2	57080448 (100 %)	54403376 (95.3 %)	2443831 (4.3 %)	51959545 (91.0 %)
mock7D ISE42_3	52595648 (100 %)	49952487 (95.0 %)	1758221 (3.3 %)	48194266 (91.6 %)
AMF7D ISE42_1	61369528 (100 %)	53396511 (87.0 %)	1919314 (3.1 %)	51477197 (83.9 %)
AMF7D ISE42_2	53260916 (100 %)	48990508 (92.0 %)	1561836 (2.9 %)	47428672 (89.1 %)
AMF7D ISE42_3	48390380 (100 %)	44962125 (92.9 %)	2236593 (4.6 %)	42725532 (88.3 %)
mock14D TT8_1	44041358 (100 %)	41246575 (93.7 %)	5484950 (12.5 %)	35761625 (81.2 %)
mock14D TT8_2	39566824 (100 %)	32513303 (82.2 %)	2650399 (6.7 %)	29862904 (75.5 %)
mock14D TT8_3	43030582 (100 %)	39977489 (92.9 %)	5527486 (12.9 %)	34450003 (80.1 %)
AMF14D TT8_1	44935916 (100 %)	38839908 (86.4 %)	2229789 (5.0 %)	36610119 (81.5 %)
AMF14D TT8_2	46755688 (100 %)	36546357 (78.2 %)	3742223 (8.0 %)	32804134 (70.2 %)
AMF14D TT8_3	34965366 (100 %)	30865312 (88.3 %)	1820174 (5.2 %)	29045138 (83.1 %)
mock14D ISE42_1	35084668 (100 %)	33203498 (94.6 %)	1648358 (4.7 %)	31555140 (90.0 %)
mock14D ISE42_2	42346422 (100 %)	40155442 (94.8 %)	2111547 (5.0 %)	38043895 (89.8 %)
mock14D ISE42_3	43055688 (100 %)	40947120 (95.1 %)	2252788 (5.2 %)	38694332 (89.9 %)
AMF14D ISE42_1	36494082 (100 %)	33018067 (90.5 %)	1698588 (4.7 %)	31319479 (85.8 %)
AMF14D ISE42_2	40322494 (100 %)	36022909 (89.3 %)	1377767 (3.4 %)	34645142 (85.9 %)
AMF14D ISE42_3	42785656 (100 %)	40233839 (94.0 %)	1551958 (3.6 %)	38681881 (90.4 %)

Supplementary Table 4. Biological processes upregulated in response to AM symbiosis.

ID	Description	Count[1]	p.adjust[2]
	TT8 7 DAT		
GO:0008037	cell recognition	5	0.029
GO:0048544	recognition of pollen	5	0.029
GO:0009875	pollen-pistil interaction	5	0.029
GO:0009856	pollination	6	0.029
GO:0044706	multi-multicellular organism process	6	0.029
GO:0006855	drug transmembrane transport	5	0.046
	ISE42 7 DAT		
GO:0046777	protein autophosphorylation	26	0.001
GO:2000022	regulation of jasmonic acid mediated signaling pathway	12	0.001
GO:0009611	response to wounding	20	0.011
GO:0009607	response to biotic stimulus	55	0.014
GO:0031407	oxylipin metabolic process	12	0.015
GO:0031408	oxylipin biosynthetic process	12	0.015
GO:0015706	nitrate transport	8	0.015
GO:0017001	antibiotic catabolic process	38	0.015
GO:0042743	hydrogen peroxide metabolic process	37	0.015
GO:0042744	hydrogen peroxide catabolic process	36	0.015
GO:0010200	response to chitin	7	0.015
GO:0007166	cell surface receptor signaling pathway	24	0.015
GO:0031347	regulation of defense response	24	0.015
GO:0002239	response to oomycetes	12	0.021
GO:0010167	response to nitrate	8	0.029
GO:0016999	antibiotic metabolic process	48	0.032
GO:0080134	regulation of response to stress	33	0.036
GO:0072593	reactive oxygen species metabolic process	39	0.048
GO:0002229	defense response to oomycetes	11	0.048
GO:1990748	cellular detoxification	44	0.048
	TT8 14 DAT		
GO:0019748	secondary metabolic process	11	<0.001
GO:0009698	phenylpropanoid metabolic process	9	<0.001
GO:0009808	lignin metabolic process	6	0.003
GO:0044550	secondary metabolite biosynthetic process	6	0.004
GO:0009699	phenylpropanoid biosynthetic process	5	0.004
GO:0009800	cinnamic acid biosynthetic process	3	0.004
GO:0009803	cinnamic acid metabolic process	3	0.004
GO:0006559	L-phenylalanine catabolic process	3	0.012
GO:1902222	erythrose 4-phosphate/phosphoenolpyruvate family amino acid catabolic process	3	0.012
GO:0009074	aromatic amino acid family catabolic process	3	0.034
GO:0006558	L-phenylalanine metabolic process	3	0.034
GO:1902221	erythrose 4-phosphate/phosphoenolpyruvate family amino acid metabolic process	3	0.034
	ISE42 14 DAT		
GO:0046271	phenylpropanoid catabolic process	3	0.018
GO:0046274	lignin catabolic process	3	0.018
GO:0019748	secondary metabolic process	4	0.022
GO:0009808	lignin metabolic process	3	0.025

[1] Number of genes affected in corresponding terms
[2] Adjusted p value.

Supplementary Table 5. Biological processes downregulated in response to AM symbiosis.

ID	Description	Count[1]	p.adjust[2]
	TT8 7 DAT		
GO:0016036	cellular response to phosphate starvation	4	<0.001
GO:0070417	cellular response to cold	3	<0.001
GO:0009267	cellular response to starvation	4	0.001
GO:0031669	cellular response to nutrient levels	4	0.001
GO:0042594	response to starvation	4	0.001
GO:0031668	cellular response to extracellular stimulus	4	0.001
GO:0071496	cellular response to external stimulus	4	0.001
GO:0031667	response to nutrient levels	4	0.002
GO:0009991	response to extracellular stimulus	4	0.003
GO:0006071	glycerol metabolic process	2	0.042
GO:0019400	alditol metabolic process	2	0.042
	ISE42 7 DAT		
GO:0006833	water transport	10	<0.001
GO:0042044	fluid transport	10	<0.001
GO:0009408	response to heat	17	<0.001
GO:0030001	metal ion transport	30	<0.001
GO:0009266	response to temperature stimulus	23	0.004
GO:0010035	response to inorganic substance	25	0.017
GO:0042542	response to hydrogen peroxide	7	0.046
GO:0043269	regulation of ion transport	6	0.048
	TT8 14 DAT		
GO:0042542	response to hydrogen peroxide	4	0.011
	ISE42 14 DAT		
GO:0045491	xylan metabolic process	1	0.045
GO:0010410	hemicellulose metabolic process	1	0.045
GO:0010383	cell wall polysaccharide metabolic process	1	0.045
GO:0044036	cell wall macromolecule metabolic process	1	0.045
GO:0000272	polysaccharide catabolic process	1	0.045
GO:0098869	cellular oxidant detoxification	1	0.045
GO:0016052	carbohydrate catabolic process	1	0.045
GO:1990748	cellular detoxification	1	0.045
GO:0097237	cellular response to toxic substance	1	0.045
GO:0098754	detoxification	1	0.045
GO:0009636	response to toxic substance	1	0.048
GO:0006979	response to oxidative stress	1	0.048

[1] Number of genes affected in corresponding terms
[2] Adjusted *p* value.

Supplementary Table 6. Expression of AM symbiosis-conserved genes in TT8 and ISE42 under drought stress conditions.

Gene ID	Gene annotation	7 DAT$		14 DAT	
		TT8	ISE42	TT8	ISE42
SETIT_034161mg	Cation channel (Castor)	-0.01	0.10	0.12	0.06
SETIT_029334mg	Receptor-like kinase (DMI2)	0.51	0.22	0.16	-0.05
SETIT_021787mg	Calcium/calmodulin-dependent protein kinase (DMI3)	-0.09	2.09*	-0.10	-0.25
SETIT_004288mg	Phosphate transporter (PHT1;9)	5.28*	7.87*	ND	ND
SETIT_008637mg	DNA-binding transcriptional activator (CYCLOPS)	ND	2.30*	ND	ND
SETIT_015555mg	ABCG half-ABC transporter (STR1)	0.21	2.55*	ND	-0.39
SETIT_003786mg	ABCG half-ABC transporter (STR2)	0.61	4.58*	ND	-0.47
SETIT_012264mg	ABC transporter (ABCB12)	1.08	6.48*	ND	ND
SETIT_015752mg	ABC transporter (ABCB20)	ND	5.05*	ND	ND
SETIT_035002mg	MSP and ANK repeat-containing protein (VAPYRIN)	0.73	0.72	0.60	0.30
SETIT_035307mg	Glycerol-3-phosphate acyl transferase (RAM2)	ND	11.32*	ND	ND
SETIT_027708mg	GRAS transcription factor (RAM1)	9.44*	7.90*	ND	ND
SETIT_000959mg	GRAS transcription factor (RAD1)	0.69	2.67*	1.67*	0.29
SETIT_021890mg	GRAS transcription factor (TF72)	1.80	3.17*	ND	ND
SETIT_003998mg	acyl-(acyl carrier protein) thioesterase (FatM)	ND	3.88*	ND	ND
SETIT_015320mg	protein binding protein (Exo70I)	0.09	4.39*	ND	ND
SETIT_024947mg	ammonium transporter (AMT2;1)	-0.22	0.68	0.28	-0.26
SETIT_004697mg	ammonium transporter (AMT3;1)	0.79	3.11*	1.45*	0.19
SETIT_026160mg	oligopeptide transporter	0.52	-1.21*	0.01	-0.12
SETIT_004857mg	nitrate transporter (ntr1)	0.41	4.51*	-0.16	-0.10
SETIT_038848mg	AP2 domain protein (AP2c)	ND	3.00*	ND	ND
SETIT_032213mg	AP2 domain protein (AP2d)	ND	6.84*	ND	ND
SETIT_001909mg	AP2 domain protein (AP2)	ND	7.77*	ND	ND
SETIT_022186mg	AP2 domain protein (AP2)	0.32	3.20*	-0.44	-0.73
SETIT_007857mg	AP2 domain protein (AP2)	ND	9.36*	ND	ND
SETIT_023636mg	CCAAT-binding transcription factor (CBF1)	0.66	2.36*	ND	0.53
SETIT_039424mg	Phytocyanin (BCP1)	-0.57	ND	ND	ND
SETIT_039030mg	plastocyanin-like domain protein	ND	9.45*	ND	ND
SETIT_039259mg	plastocyanin-like domain protein	ND	8.04*	ND	ND
SETIT_020176mg	plastocyanin-like domain protein	1.05	0.10	0.37	-1.09
SETIT_007296mg	plastocyanin-like domain protein	0.70	-1.30*	ND	ND
SETIT_038020mg	plastocyanin-like domain protein	0.24	4.00*	0.64	1.65
SETIT_011928mg	subtilisin inhibitor	ND	4.72*	ND	ND
SETIT_023913mg	subtilisin inhibitor	ND	7.29*	ND	ND

$ * indicates genes that were significantly upregulated or downregulated in response to AM symbiosis. ND indicates that the transcript was not detected in the transcriptome dataset.

Supplementary Table 6. Expression of AM symbiosis-conserved genes in TT8 and ISE42 under drought stress conditions (continued).

Gene ID	Gene	7 DAT[$]		14 DAT	
		TT8	ISE42	TT8	ISE42
SETIT_019796mg	subtilisin inhibitor	ND	13.57*	ND	ND
SETIT_033231mg	class III chitinase	2.74	6.84*	ND	ND
SETIT_021498mg	class III chitinase	0.99	1.76*	0.51	0.26
SETIT_002402mg	class III chitinase	2.47	5.42*	0.50	0.07
SETIT_004962mg	Protein kinase (kin4)	-0.36	3.98*	0.10	-0.99
SETIT_028049mg	Protein kinase (kin3)	ND	6.40*	ND	ND
SETIT_006522mg	Protein kinase (kin2)	ND	7.62*	ND	ND
SETIT_039206mg	late embryogenesis abundant (lea)	ND	7.91*	ND	ND
SETIT_023002mg	late embryogenesis abundant (lea)	0.60	2.05*	0.81	0.78
SETIT_010999mg	late embryogenesis abundant (lea)	0.26	1.00*	-0.01	0.35
SETIT_023221mg	late embryogenesis abundant (lea)	0.22	-1.62*	0.23	0.16
SETIT_032400mg	germin-like protein	ND	12.10*	ND	ND
SETIT_033039mg	germin-like protein	ND	8.85*	ND	ND
SETIT_038965mg	Nod-factor receptor 5	2.40*	-0.23	ND	ND
SETIT_028233mg	heparan-alpha-glucosaminide N-acetyltransferase	-0.09	1.71*	-0.12	0.16
SETIT_017532mg	Protein of unknown function	-0.21	-0.47	0.20	0.63
SETIT_017497mg	GDSL-like lipase/acylhydrolase	-0.32	-0.62	0.16	-0.12
SETIT_032023mg	DnaJ domain protein	0.56	1.66*	ND	ND
SETIT_032512mg	DnaJ domain protein	ND	5.49*	ND	ND
SETIT_029260mg	9-cis-epoxycarotenoid dioxygenase (CCD8b)	0.11	0.80	-0.27	ND
SETIT_012330mg	citrate-binding protein (CBP1)	0.08	-0.23	ND	ND
SETIT_039331mg	cytochrome protein b561 CYTb1)	0.02	2.80*	-0.13	-0.45
SETIT_038937mg	cytochrome P450 (CYT733A1)	0.32	4.84*	-0.43	0.18
SETIT_024818mg	short-chain dehydrogenase/reductase (DHY1)	-0.43	2.60*	ND	0.45
SETIT_038748mg	glutathione S-transferase tau (GST1)	-0.50	0.01	-0.49	0.06
SETIT_029284mg	serine-threonine protein kinase (KIN5)	-0.20	0.41	0.05	-0.23
SETIT_023557mg	heavy-metal-associated domain protein (HMAD1)	0.29	-0.11	0.01	-0.04
SETIT_039811mg	ammonium transporter (AMT2;4)	ND	4.62*	ND	ND
SETIT_027981mg	GRAS family transcription factor (DIP1b)	0.40	-1.94*	ND	ND

[$] * indicates genes that were significantly upregulated or downregulated in response to AM symbiosis. ND indicates that the transcript was not detected in the transcriptome dataset.

Supplementary Table 7. Differentially expressed *AP2/ERF* genes in TT8 and ISE42 in response to AM symbiosis during drought stress conditions.

Gene ID	7 DAT[$]		14 DAT	
	TT8	ISE42	TT8	ISE42
SETIT_007857mg	ND	9.36*	ND	ND
SETIT_001909mg	ND	7.77*	ND	ND
SETIT_008428mg	ND	7.64*	ND	ND
SETIT_032351mg	ND	6.52*	ND	ND
SETIT_018144mg	0.48	5.47*	0.39	0.72
SETIT_033237mg	-1.28	4.22*	0.56	ND
SETIT_036647mg	0.10	3.82*	0.55	0.66
SETIT_022186mg	0.32	3.20*	-0.44	-0.73
SETIT_038848mg	ND	3.00*	ND	ND
SETIT_024576mg	-0.77	2.99*	ND	0.67
SETIT_002247mg	0.20	2.93*	-0.20	0.50
SETIT_018039mg	1.08	2.93	0.23	2.10*
SETIT_017790mg	0.17	2.76*	-0.01	-0.41
SETIT_031079mg	ND	2.60*	ND	ND
SETIT_023329mg	-0.55	2.42*	-0.81	1.44*
SETIT_002010mg	-0.40	2.40*	-0.35	-0.06
SETIT_003053mg	0.49	2.38*	-0.07	-0.19
SETIT_002845mg	0.99	2.24*	-0.46	1.90*
SETIT_026709mg	-0.55	2.09*	0.06	-0.25
SETIT_017941mg	-0.15	2.07*	0.23	-0.08
SETIT_040030mg	ND	2.04*	ND	ND
SETIT_036821mg	0.74	1.92*	0.01	0.93
SETIT_031946mg	-0.07	1.89*	0.65	-0.25
SETIT_018188mg	ND	1.68*	ND	ND
SETIT_036615mg	-0.55	1.62*	0.14	-0.25
SETIT_024479mg	1.74	1.61*	-0.47	0.17
SETIT_030810mg	ND	1.51*	ND	ND
SETIT_017568mg	0.07	1.33*	0.11	-0.05
SETIT_030088mg	0.02	1.17*	0.13	0.19
SETIT_039427mg	-0.15	1.06*	-0.74	0.81
SETIT_004222mg	-1.25*	0.55	-0.21	-0.65
SETIT_021952mg	-0.02	0.35	-1.27*	0.53
SETIT_021959mg	-0.73	0.16	-1.20*	0.14
SETIT_030856mg	1.40	0.04	0.91	1.70*
SETIT_028170mg	-0.73	-0.71	-1.19*	0.17
SETIT_012254mg	-0.06	-1.03*	-0.21	-0.05
SETIT_013986mg	-0.50	-1.10*	0.14	-0.38
SETIT_022788mg	0.01	-1.67*	0.18	0.47
SETIT_015394mg	0.00	-2.38*	0.36	-0.26
SETIT_018222mg	ND	-2.80*	ND	ND
SETIT_036893mg	-0.60	-3.64*	0.37	-0.10
SETIT_032213mg	ND	6.84*	ND	ND
SETIT_002846mg	ND	5.18*	-0.28	-0.68
SETIT_002010mg	-0.40	2.40*	-0.35	-0.06
SETIT_011005mg	1.61	0.69	0.41	2.18*
SETIT_031001mg	0.85	0.49	0.85	1.84*
SETIT_030998mg	0.70	0.27	0.38	1.31*
SETIT_030869mg	0.97	0.21	1.32*	1.70*
SETIT_018262mg	1.71	ND	-0.15	1.59*

[$] * indicates genes that were significantly upregulated or downregulated in response to AM symbiosis. ND indicates that the transcript was not detected in the transcriptome dataset.

Supplementary Table 8. Differentially expressed *NAC* genes in TT8 and ISE42 in response to AM symbiosis during drought stress conditions.

Gene ID	7 DAT[$]		14 DAT		Gene ID	7 DAT		14 DAT	
	TT8	ISE42	TT8	ISE42		TT8	ISE42	TT8	ISE42
SETIT_004195mg	ND	6.18*	ND	ND	SETIT_006994mg	1.64	1.94*	ND	0.27
SETIT_026707mg	ND	4.73*	ND	ND	SETIT_001354mg	0.56	1.93*	0.44	0.65
SETIT_036301mg	-0.63	4.40*	0.86	-1.73	SETIT_010410mg	-1.28*	1.88*	0.30	-0.25
SETIT_002586mg	0.44	4.15*	0.19	-0.52	SETIT_010417mg	0.24	1.79*	0.07	0.01
SETIT_022966mg	1.97	3.94*	ND	ND	SETIT_028024mg	ND	1.51*	ND	ND
SETIT_032881mg	-0.38	3.86*	ND	0.51	SETIT_030526mg	0.40	1.36*	0.24	-0.25
SETIT_002386mg	-0.09	3.44*	0.05	-0.03	SETIT_006975mg	0.54	1.17*	0.96	-0.06
SETIT_030752mg	1.18	3.13*	0.34	0.23	SETIT_036757mg	0.99	1.09*	1.13	-0.05
SETIT_017393mg	ND	2.86*	ND	0.07	SETIT_024889mg	-0.17	1.09*	0.03	-0.36
SETIT_012740mg	0.35	2.40*	ND	ND	SETIT_004848mg	-0.03	1.00*	0.81	0.07
SETIT_010680mg	0.53	2.32*	ND	ND	SETIT_032676mg	-0.72	0.43	1.31*	-0.11
SETIT_036241mg	-0.31	2.20*	0.32	0.07	SETIT_014180mg	ND	-1.05*	ND	ND
SETIT_036265mg	0.31	2.15*	0.60	0.28	SETIT_036535mg	2.33	-1.25*	ND	ND
SETIT_010735mg	0.62	2.08*	ND	-0.20	SETIT_017567mg	-0.13	-1.26*	0.14	-0.47
SETIT_015514mg	0.93	2.06*	ND	ND	SETIT_006565mg	1.17	-1.36*	ND	2.94*
SETIT_019775mg	-0.65	2.03*	ND	ND	SETIT_010799mg	ND	-1.55*	ND	ND
SETIT_001021mg	0.49	2.03*	0.43	0.43	SETIT_011079mg	-0.25	-1.58*	-0.18	-0.33
SETIT_026342mg	0.22	1.96*	0.42	0.20	SETIT_017967mg	-0.34	-2.50*	-0.27	-0.05

[$] * indicates genes that were significantly upregulated or downregulated in response to AM symbiosis. ND indicates that the transcript was not detected in the transcriptome dataset.

Supplementary Table 9. Differentially expressed genes associated with calcium signaling pathways in response to AM symbiosis.

Gene ID	Gene	7 DAT$		14 DAT	
		TT8	ISE42	TT8	ISE42
SETIT_029431mg	calcium-dependent protein kinase (SiCDPK8)	0.306	3.078*	0.114	-0.278
SETIT_034743mg	calcium-dependent protein kinase (SiCDPK26)	0.379	2.712*	0.444	0.062
SETIT_034847mg	calcium-dependent protein kinase (SiCDPK27)	0.372	2.354*	0.509	-0.161
SETIT_0263202mg	calcium-dependent protein kinase (SiCDPK23)	-0.097	2.294*	0.381	0.108
SETIT_009748mg	calcium-dependent protein kinase (SiCDPK19)	0.247	2.048*	0.210	0.239
SETIT_0263201mg	calcium-dependent protein kinase (SiCDPK23)	0.044	1.964*	0.201	0.103
SETIT_016899mg	calcium-dependent protein kinase (SiCDPK1)	0.194	1.826*	0.327	-0.013
SETIT_029530mg	calcium-dependent protein kinase (SiCDPK5)	0.026	1.636*	0.238	-0.130
SETIT_040704mg	calcium-dependent protein kinase (SiCDPK29)	-0.199	1.576*	0.461	0.183
SETIT_021672mg	calcium-dependent protein kinase (SiCDPK10)	0.152	1.445*	-0.115	0.304
SETIT_016803mg	calcium-dependent protein kinase (SiCDPK2)	0.206	1.193*	0.111	0.216
SETIT_029046mg	CSC1-like protein	0.355	3.100*	0.393	-0.473
SETIT_034294mg	CSC1-like protein	0.029	2.732*	0.334	1.288*
SETIT_024448mg	CSC1-like protein	-0.414	2.317*	ND	0.248
SETIT_021277mg	CSC1-like protein	-0.202	2.113*	0.191	-0.198
SETIT_002788mg	calcium-binding protein	0.820	2.989*	ND	ND
SETIT_003742mg	calcium-binding protein	ND	2.979*	ND	ND
SETIT_004282mg	calcium-binding protein	0.016	2.633*	-0.546	0.172
SETIT_010908mg	calcium-binding protein	0.983	1.922*	-0.093	0.541
SETIT_011148mg	calcium-binding protein	0.018	1.808*	0.193	-0.776
SETIT_023228mg	calcium-binding protein	0.778	1.823*	0.036	-0.275
SETIT_005109mg	calcium-binding protein	0.837	1.770*	0.511	1.333*
SETIT_039896mg	calcium-binding protein	0.664	1.600*	0.444	0.002
SETIT_010743mg	calcium-binding protein	-0.262	1.237*	0.333	-0.416
SETIT_026676mg	calcium-binding protein	0.012	1.157*	0.134	0.054
SETIT_009077mg	calcium-binding protein	0.618	-0.593	0.927	1.167*
SETIT_023383mg	calcium-binding protein	0.644	1.225*	0.650	1.265*
SETIT_039755mg	calcium-binding protein	-0.433	-1.280*	0.116	-0.196
SETIT_004028mg	calcium-binding protein	1.421	-2.762*	ND	ND

$ * indicates genes that were significantly upregulated or downregulated in response to AM symbiosis. ND indicates that the transcript was not detected in the transcriptome dataset.

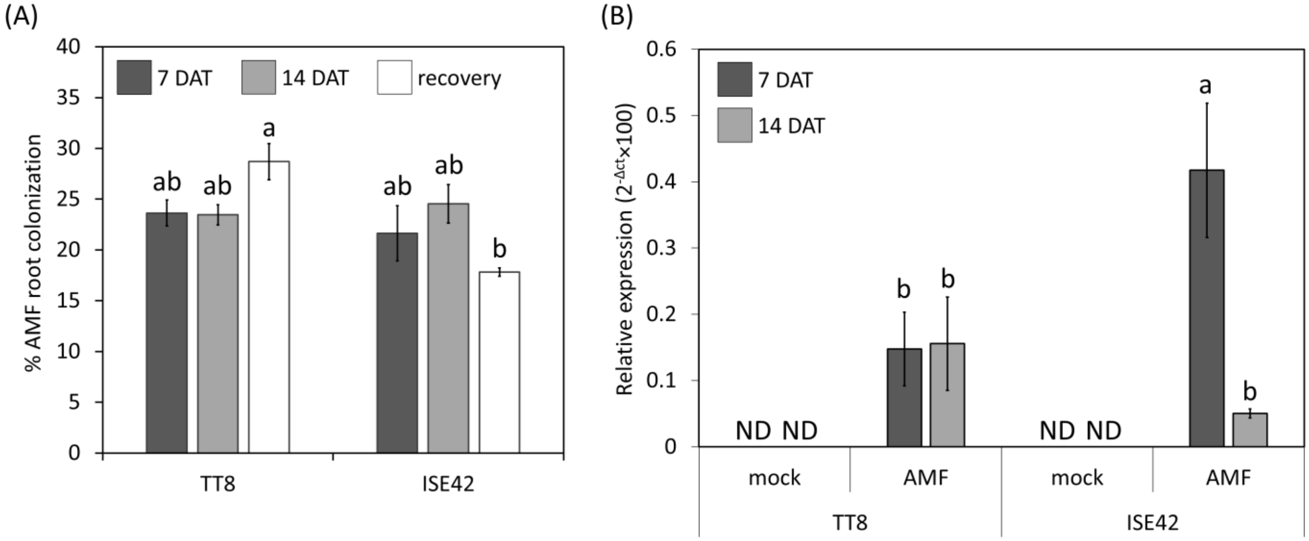

Supplementary Figure 1. AMF colonization efficiency in TT8 and ISE42 during drought stress and after water resupply. (A) Percentage of AMF colonization efficiency (N=5). (B) Relative expression of *C. etunicatum* β*-Tubulin* gene (N=3). Error bars represent the standard error of the mean. Data were analyzed using ANOVA (p<0.05). Different letters above the bars indicate statistically significant differences.

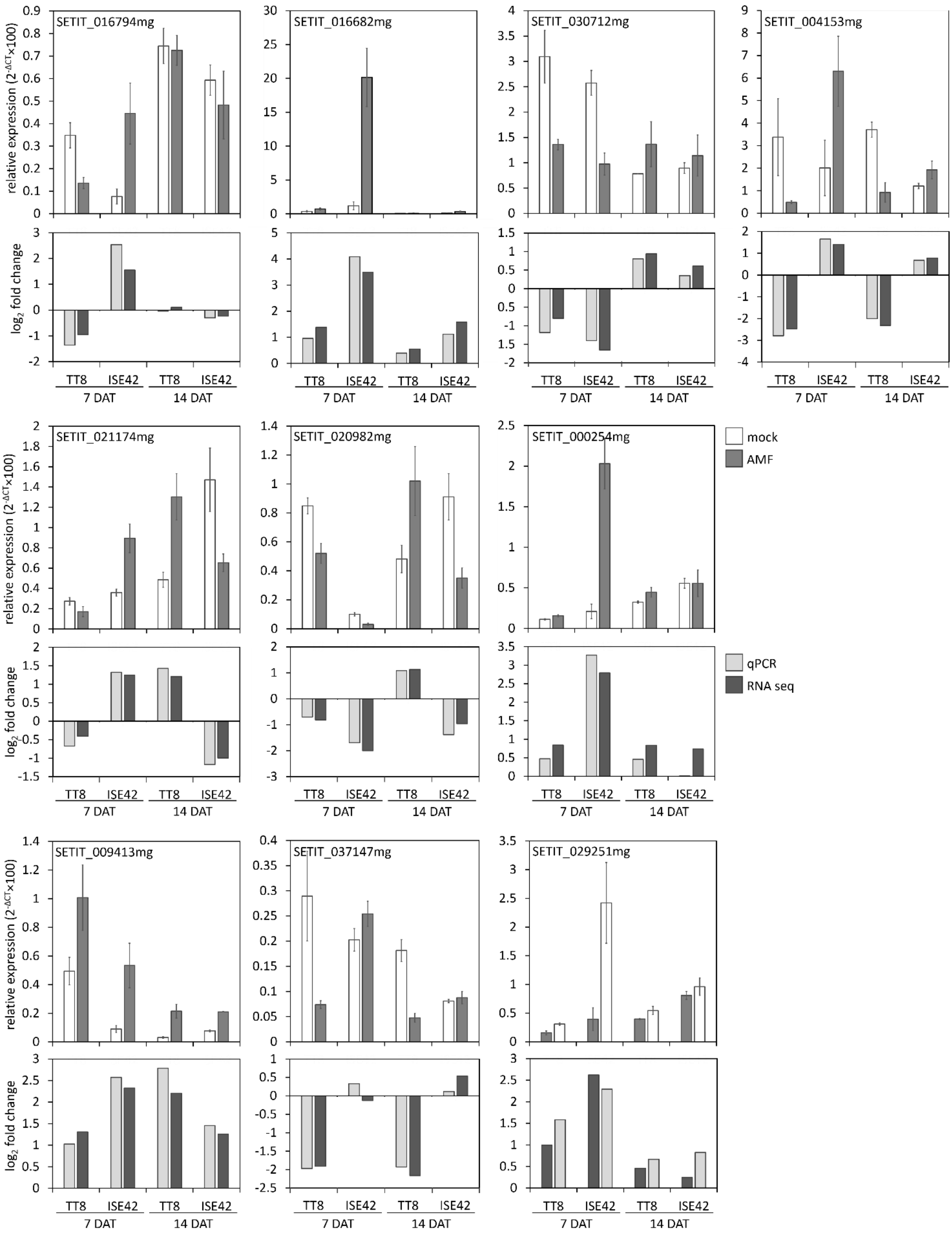

Supplementary Figure 2. qRT-PCR validation of selected DEGs identified by RNA-seq. N=3. Error bars represent standard error of the mean.

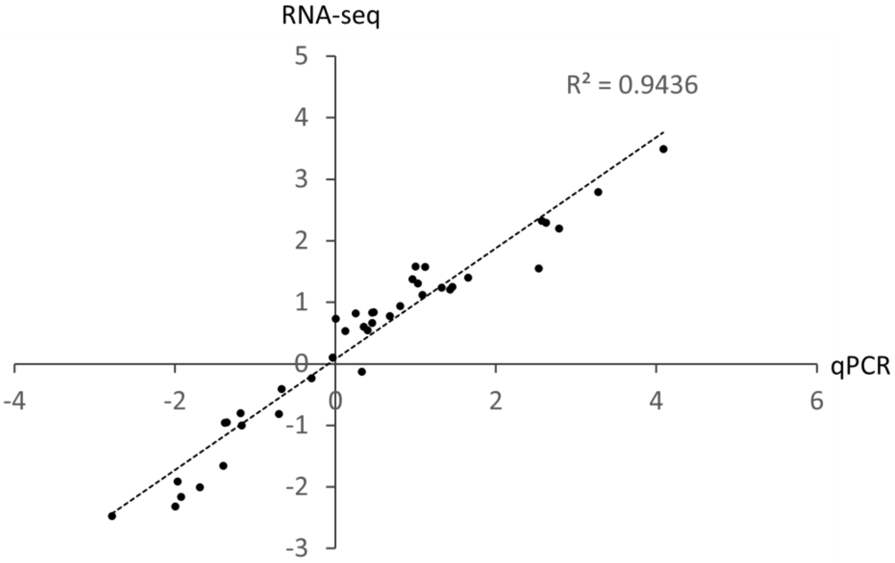

Supplementary Figure 3. Correlation of gene expression fold changes as determined by RNA sequencing and validated by qRT-PCR analyses.

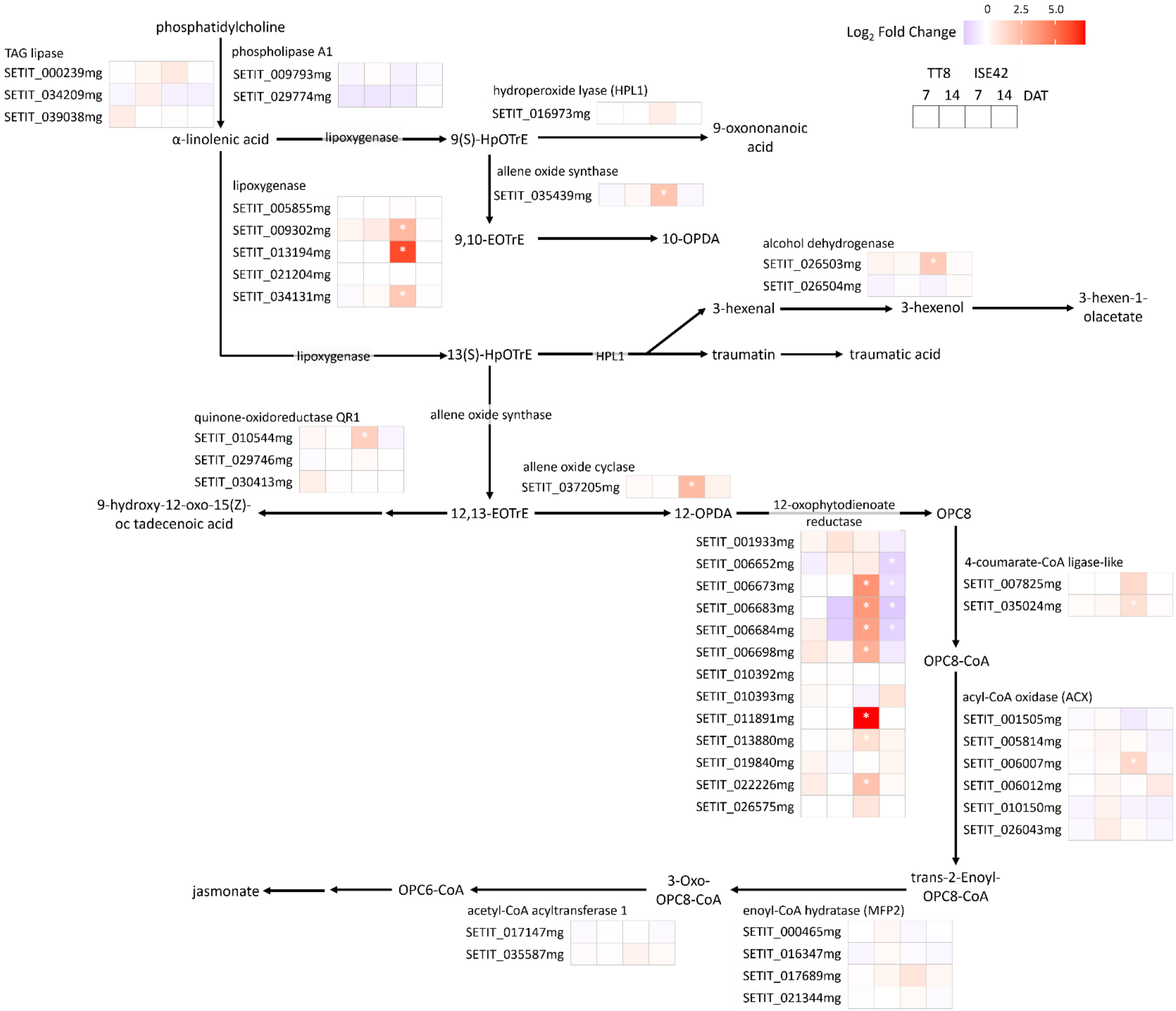

Supplementary Figure 4. Effects of AM symbiosis on the expression of genes involved in α-linolenic acid metabolism in TT8 and ISE42.
